# Protective and Adverse Roles of DDX3X in Different Cell Types in Nonalcoholic Steatohepatitis Progression

**DOI:** 10.34133/research.0275

**Published:** 2023-12-08

**Authors:** Suzhen Yang, Lin Zhou, Tianming Zhao, Hanlong Zhu, Tingting Luo, Kang Jiang, Xiaoxiao Shi, Chunyan Chen, Han Zhang, Si Zhao, Xiaoping Zou, Yuzheng Zhuge, Fangyu Wang, Lei Wang, Mingzuo Jiang, Bing Xu

**Affiliations:** ^1^ Department of Gastroenterology, the Affiliated Drum Tower Hospital of Nanjing University Medical School, Jiangsu, Nanjing, 210002, China.; ^2^ Department of Gastroenterology and Hepatology, the Affiliated Jinling Hospital of Nanjing University Medical School, Jiangsu, Nanjing, 210002, China; ^3^ School of Medicine, Northwest University, Shaanxi, Xi’an, 710069, China.; ^4^ Department of Gastroenterology, Nanjing Drum Tower Hospital, Chinese Academy of Medical Sciences & Peking Union Medical College, Jiangsu, Nanjing, 210008, China.

## Abstract

Persistent hepatic cellular metabolic stress and liver inflammatory stimuli are key signatures of nonalcoholic steatohepatitis (NASH). DDX3X is a vital molecule involved in cell fate decisions in both pro-survival stress granule (SG) and pro-death NOD-like receptor family pyrin domain containing 3 (NLRP3) inflammasome assembly in response to stress signals. However, the role of DDX3X in NASH remains unclear. We characterized the cell type-specific roles of DDX3X in NASH. Human liver tissues from NASH patients and normal control subjects were collected to assess DDX3X expression and distribution. Nutritional steatohepatitis models were constructed by feeding macrophage-specific DDX3X knockout (DDX3X^ΔMφ^), hepatocyte-specific DDX3X knockout (DDX3X^Δhep^), and wild-type control (DDX3X^fl/fl^) mice a high-fat and high-cholesterol (HFHC) diet, a methionine- and choline-deficient (MCD) diet, and a high-fat/high-iron/high-fructose/high-cholesterol, low-methionine, and choline-deficient (HFHIHFHC-MCD) diet. The study demonstrated that DDX3X was predominantly expressed in macrophages and hepatocytes in control liver tissues, and its expression was down-regulated in patients or mice with NASH. Compared to DDX3X^fl/fl^ littermates, DDX3X^ΔMφ^ mice showed improved liver histology in nutritional steatohepatitis models. Loss of macrophage DDX3X inhibited NLRP3 inflammasome-mediated pyroptosis, causing anti-inflammatory M2 polarization and alleviating hepatocyte steatohepatitic changes. DDX3X^Δhep^ mice developed marked steatohepatitis in multiple nutritional steatohepatitis models compared to DDX3X^fl/fl^ littermates. DDX3X-deleted hepatocytes showed impaired SG assembly, leading to increased sensitivity and intolerance to metabolic stimulation and resultant steatohepatitis. In conclusion, DDX3X plays opposite roles in different cell types during the progression of NASH. A better understanding of the cell-specific differences in the crosstalk between SG formation and NLRP3 activation is crucial for developing prospective targeted DDX3X inhibitors for the treatment of NASH.

## Introduction

Nonalcoholic fatty liver disease (NAFLD) comprises a multistep clinicopathological spectrum of chronic liver disorders. Nonalcoholic steatohepatitis (NASH) is a more severe form of NAFLD marked by necro-inflammation and hepatocyte ballooning [1,2]. Continuous hepatic cellular metabolic stress and prolonged liver inflammatory insults are believed to be the key signatures of steatohepatitis, which perpetuates hepatocellular injury and enables cells to perish, thus promoting liver fibrosis [3–5]. However, the exact pathogenic network that interprets stress signals in the initiation and propagation of steatohepatitis remains incompletely elucidated.

DEAD-box helicase 3, X-linked (*Ddx3x*), a member of the DEAD-box family, has been reported to regulate lipid homeostasis during hepatitis C virus (HCV)-induced steatosis [6]. Recent study reported that hepatic DDX3X relieved high-fat diet (HFD)-induced lipid metabolism disorder and liver steatosis [7]. However, mice fed an HFD develop NAFLD but rarely NASH. Approximately 25% of NAFLD cases progress to NASH, and NASH-related complications are the major factors affecting disease outcomes in NAFLD patients [8]. It is important to illustrate the pathogenesis of steatosis-to-NASH progression. Our group was one of the first to document that gasdermin D (GSDMD)-driven pyroptosis, a downstream executor of several inflammasomes, is the key inflammatory link between NAFLD and NASH [9]. DDX3X has been identified as a central decision molecule in stress granules (SGs) and NOD-like receptor family pyrin domain containing 3 (NLRP3) inflammasome assembly in response to cellular stress signals in bone marrow-derived macrophages (BMDMs) [10,11]. Both SGs and NLRP3 inflammasomes are intracellular complexes that assemble under cell stress stimuli, but they cause opposite cellular fates [11]. SG formation helps cells survive under cellular stress [12,13]. However, activation of inflammasomes is a powerful trigger of inflammation, which leads to execution of pyroptosis and induces interleukin-1β (IL-1β) and IL-18 secretion [14–16]. Pyroptosis is an important inflammatory mediator, and uncontrolled pyroptosis may cause cell death and disease progression [17–19]. Pyroptosis has been reported to be involved in the pathomechanism of liver warm ischemia–reperfusion injury (IRI), alcoholic liver disease (ALD), NASH, and liver fibrosis [9,20–22]. However, the function of DDX3X on the inflammatory response and cell death in NASH progression remains unknown. In addition, the role of DDX3X in different liver cells has never been reported.

Herein, we used conditional macrophage DDX3X knockout (DDX3X^ΔMφ^) mice and conditional hepatocyte DDX3X knockout (DDX3X^Δhep^) mice to explore the cell-specific roles of DDX3X in nutritional steatohepatitis. Our data illustrate that macrophage DDX3X loss inhibits NLRP3-mediated pyroptosis and improves liver histology in nutritional steatohepatitis. Hepatocyte DDX3X deficiency mainly affects the capacity of SG assembly, leading to increased sensitivity and intolerance to metabolic stimulation and resultant steatohepatitis. Thus, a better understanding of the cell-specific differences in the crosstalk between SG formation and NLRP3 activation is essential for developing promising targeted DDX3X drugs for the treatment of NASH.

## Results

### DDX3X expression is decreased in the livers of humans and mice with NASH

**Fig. 1.**
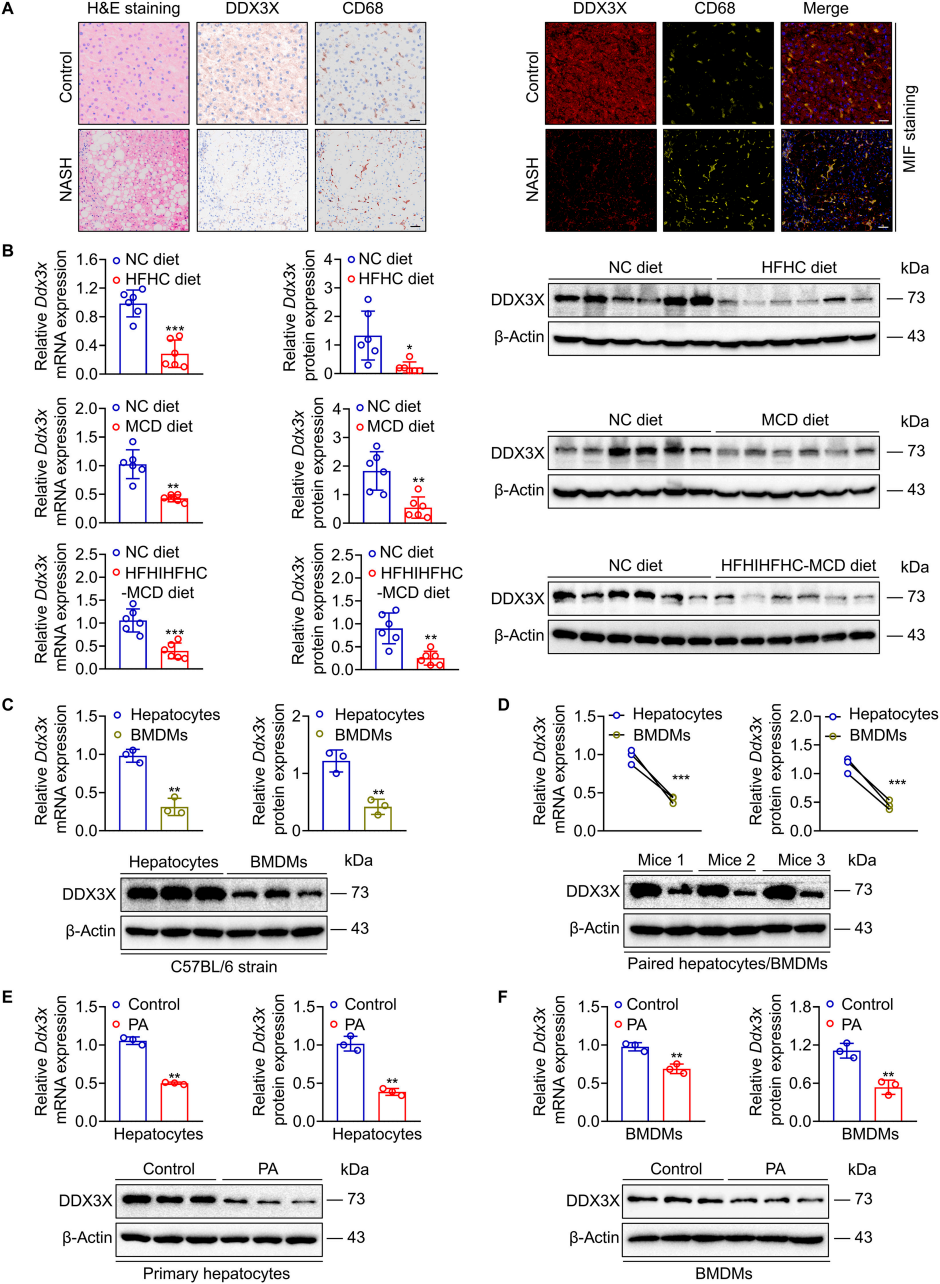
DDX3X expression is decreased in the liver tissues of humans and mice with NASH. (A) Representative MIF sections of DDX3X expression and distribution in human liver tissues. Scale bars, 50 μm. (B) mRNA and protein expression of *Ddx3x* in multiple nutritional mouse models of steatohepatitis fed an HFHC, MCD, or HFHIHFHC-MCD diet. (C and D) Expression and distribution of *Ddx3x* in primary hepatocytes and BMDMs. (E) mRNA and protein expression of *Ddx3x* in primary hepatocytes with or without PA stimulation (0.4 mM, 24 h). (F) mRNA and protein expression of *Ddx3x* in BMDMs with or without PA stimulation (0.4 mM, 24 h). *n* = 3 to 6 per group; data are indicated as the mean ± SD. *P < 0.05, **P < 0.01, ***P < 0.001.

A previous study demonstrated that the protein level of DDX3X was decreased in NAFLD patients [7]. We further analyzed the Gene Expression Omnibus (GEO) database with a larger sample size of 151 NAFLD/NASH patients and 28 controls (GSE213621) and reached the same conclusion that DDX3X expression was decreased in NASH patients (Fig. S1A). However, the specific cell distribution of DDX3X in the liver remains unclear. Therefore, we performed multiplexed immunofluorescence (MIF) and revealed that DDX3X was down-regulated in human NASH tissues compared to normal control (NC) liver tissues (Fig. 1A). More importantly, DDX3X was mainly expressed in macrophages and hepatocytes but not in CD8^+^ T cells of livers from NC subjects (Fig. 1A and Fig. S1B). Down-regulation of DDX3X mRNA and protein expression was found in multiple nutritional mouse models of steatohepatitis (Fig. 1B and Fig. S1C). The expression and distribution of DDX3X was further validated in primary hepatocytes and BMDMs. Both hepatocytes and BMDMs showed DDX3X distribution, but hepatocytes expressed higher DDX3X mRNA and protein levels than BMDMs isolated from the same mice (Fig. 1C and D).

To further investigate the expression of DDX3X in vitro, we treated primary hepatocytes and BMDMs with palmitic acid (PA), one of the most commonly used saturated fatty acid stimulants in NAFLD cell models, to stimulate the steatohepatitis environment. As shown in Fig. 1E and F, decreased DDX3X levels were further confirmed in primary hepatocytes and BMDMs treated with PA. All these results indicate that DDX3X is down-regulated in the progression of NASH.

### Macrophage DDX3X exacerbates experimental steatohepatitis

**Fig. 2.**
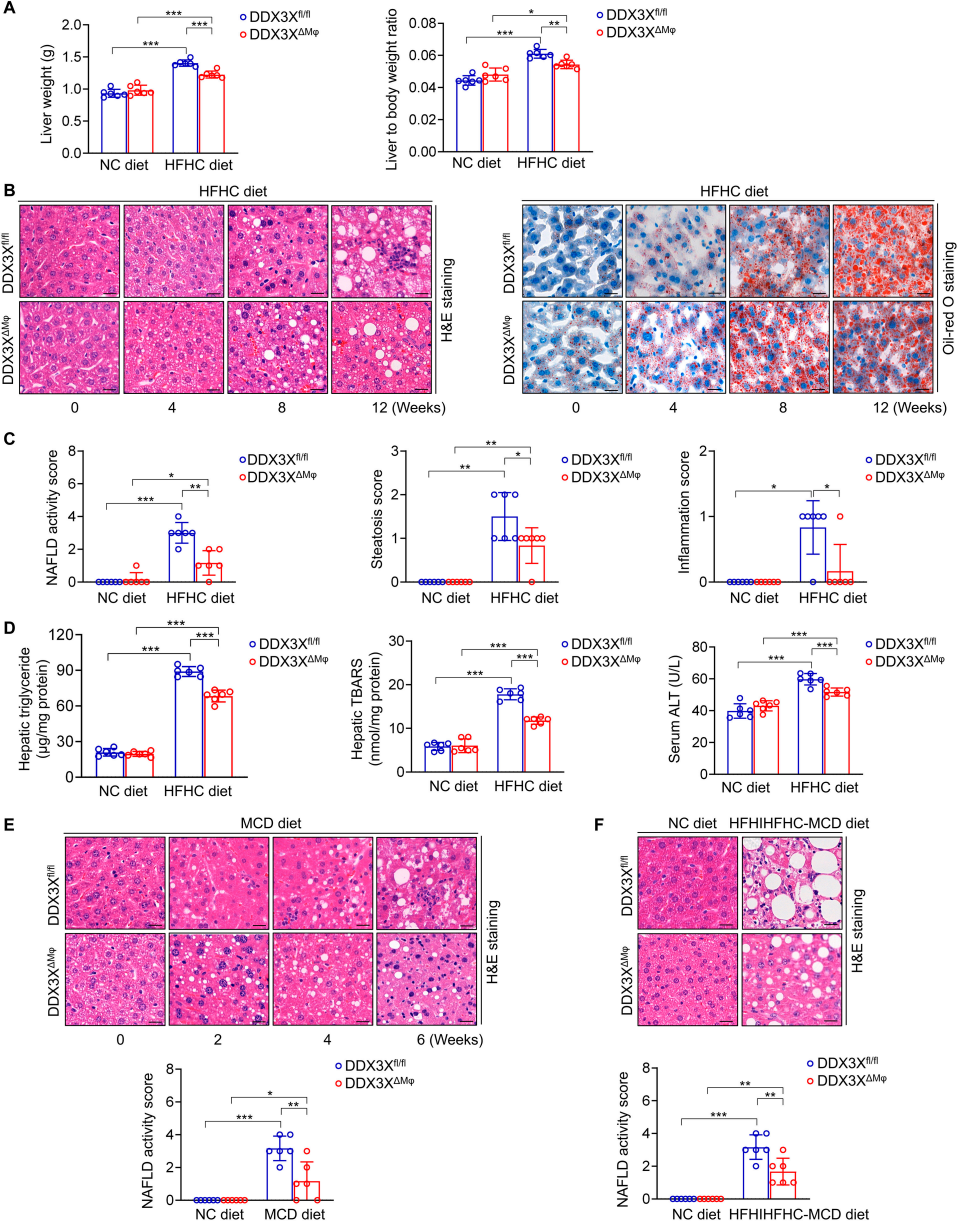
Deficiency of macrophage DDX3X attenuates experimental steatohepatitis. (A) Liver weights and liver-to-body weight ratios of DDX3X^fl/fl^ and DDX3X^ΔMφ^ mice exposed to the NC diet or HFHC diet for 12 weeks. (B) Histological evaluation of liver sections from DDX3X^fl/fl^ and DDX3X^ΔMφ^ mice fed the HFHC diet for 0, 4, 8, and 12 weeks. Scale bars, 20 μm. (C) NAFLD activity score, steatosis score, and inflammation score of liver sections were assessed in DDX3X^fl/fl^ and DDX3X^ΔMφ^ mice fed an HFHC diet for 12 weeks. (D) Hepatic TG content, liver lipid peroxidation, and serum ALT levels in DDX3X^fl/fl^ and DDX3X^ΔMφ^ mice fed an HFHC diet for 12 weeks. (E) Representative hematoxylin and eosin (H&E)-stained liver sections and NAFLD activity score of DDX3X^fl/fl^ and DDX3X^ΔMφ^ mice fed an MCD diet for 6 weeks. Scale bars, 20 μm. (F) Representative H&E-stained liver sections and NAFLD activity score of DDX3X^fl/fl^ and DDX3X^ΔMφ^ mice fed an HFHIHFHC-MCD diet for 6 weeks. Scale bars, 20 μm. *n* = 6 per group; data are expressed as the mean ± SD. *P < 0.05, **P < 0.01, ***P < 0.001.

NLRP3 in myeloid cells plays a vital role in experimental steatohepatitis [23]. A previous study demonstrated that DDX3X acted as a building block for the assembly of SGs and the NLRP3 inflammasome in murine BMDMs [11]. Therefore, we explored the effect of myeloid macrophage DDX3X on NASH. Macrophage DDX3X mRNA and protein expression were both decreased in BMDMs isolated from DDX3X^ΔMφ^ mice compared to BMDMs isolated from DDX3X^fl/fl^ mice (Fig. S2A). Compared to DDX3X^fl/fl^ mice, DDX3X^ΔMφ^ mice fed the high-fat and high-cholesterol (HFHC) diet showed markedly lower liver weights and liver-to-body weight ratios after 12 weeks of induction (Fig. 2A). Importantly, histological examination of liver sections revealed that although DDX3X^ΔMφ^ mice showed more obvious steatosis at early stages (0, 4, and 8 weeks) of HFHC diet induction, the relatively late process of steatohepatitis was significantly inhibited compared to that in DDX3X^fl/fl^ mice fed the HFHC diet for 12 weeks (Fig. 2B and C). Hepatic triglyceride (TG) content, hepatic lipid peroxide levels, and serum alanine transaminase (ALT) levels were all lower in DDX3X^ΔMφ^ mice fed the HFHC diet for 12 weeks than in DDX3X^fl/fl^ mice (Fig. 2D), suggesting that macrophage DDX3X exacerbated the development of NASH. The improved liver histology of DDX3X^ΔMφ^ mice was further confirmed in nutritional steatohepatitis models induced by methionine- and choline-deficient (MCD) diet for 6 weeks (Fig. 2E) and high-fat/high-iron/high-fructose/high-cholesterol, low-methionine, and choline-deficient (HFHIHFHC-MCD) diet for 6 weeks (Fig. 2F), suggesting that DDX3X deficiency in macrophages protected mice from steatohepatitis.

### Loss of macrophage DDX3X inhibits NLRP3 inflammasome activation and pyroptosis

**Fig. 3.**
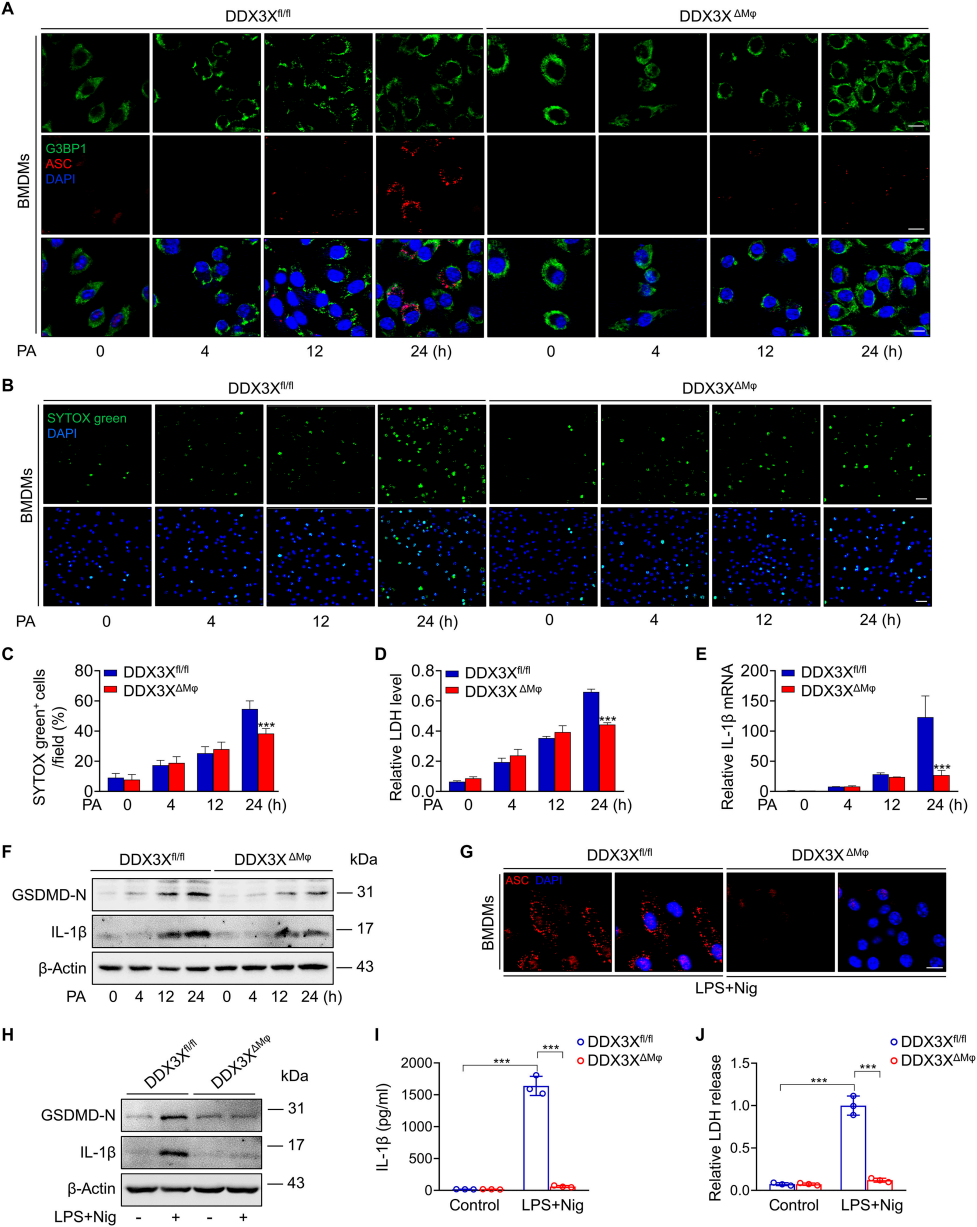
Loss of macrophage DDX3X inhibits NLRP3 inflammasome activation and pyroptosis. (A) Representative immunofluorescence images of G3BP1 and ASC in BMDMs isolated from DDX3X^fl/fl^ or DDX3X^ΔMφ^ mice with in vitro stimulation of PA (0.4 mM) for 0, 4, 12, and 24 h, respectively. Scale bars, 10 μm. (B and C) SYTOX green staining of BMDMs isolated from DDX3X^fl/fl^ or DDX3X^ΔMφ^ mice with in vitro stimulation of PA (0.4 mM) for 0, 4, 12, and 24 h, respectively. Scale bars, 25 μm. (D) Medium LDH levels and (E) IL-1β mRNA of BMDMs isolated from DDX3X^fl/fl^ or DDX3X^ΔMφ^ mice with in vitro stimulation of PA (0.4 mM) for 0, 4, 12, and 24 h, respectively. (F) GSDMD-N and IL-1β levels of BMDMs isolated from DDX3X^fl/fl^ or DDX3X^ΔMφ^ mice with stimulation of PA (0.4 mM) for 0, 4, 12, and 24 h, respectively. (G) Representative immunofluorescence images of ASC, (H) GSDMD-N and IL-1β levels, (I) IL-1β release evaluated by enzyme-linked immunosorbent assay, and (J) medium LDH levels in BMDMs isolated from DDX3X^fl/fl^ or DDX3X^ΔMφ^ mice primed with LPS for 4 h and then stimulated with the NLRP3 stimulus nigericin (Nig; 20 μM) for 45 min. Scale bars, 10 μm. *n* = 3 per group; data are expressed as the mean ± SD. ***P < 0.001.

To characterize the mechanisms of macrophage DDX3X-associated steatohepatitis, we evaluated the effect of DDX3X on governing the regulation between pro-survival and pro-death cell fate pathways in BMDMs. Coimmunoprecipitation (Co-IP) experiments showed that DDX3X could interact with both G3BP1 and NLRP3 under PA induction in BMDMs (Fig. S2B). Confocal microscopy imaging demonstrated that SG assembly (indicated by G3BP1 staining, a marker of SGs) was a relatively early event compared to NLRP3 inflammasome activation, indicated by apoptosis-associated speck-like protein containing a CARD (ASC) staining, in BMDMs treated with PA (Fig. 3A). In addition, MCC950, a specific inhibitor of the NLRP3 inflammasome, significantly inhibited GSDMD cleavage, IL-1β release, and lactate dehydrogenase (LDH) levels in DDX3X^fl/fl^ macrophages treated with PA for 24 h (Fig. S2C to E). Importantly, both SG and NLRP3 inflammasome assembly were impaired in DDX3X^ΔMφ^ macrophages compared to DDX3X^fl/fl^ macrophages (Fig. 3A). Evaluation of cell death by SYTOX Green dye staining showed a significant reduction in cell death in DDX3X^ΔMφ^ macrophages after 24 h of PA induction, despite a slightly increased level of cell death in DDX3X^ΔMφ^ macrophages at the early stages (Fig. 3B and C). Consistently, LDH, IL-1β expression, and GSDMD cleavage were all inhibited in DDX3X^ΔMφ^ macrophages compared to DDX3X^fl/fl^ macrophages treated with PA for 24 h (Fig. 3D to F). However, the NLRP3 inflammasome activator nigericin in DDX3X^ΔMφ^ macrophages failed to induce NLRP3 inflammasome activation-mediated pyroptosis (Fig. 3G to J). All these data suggested that DDX3X was indispensable in macrophage NLRP3 inflammasome activation. Our previous study demonstrated that GSDMD-driven pyroptosis acts as a critical inflammatory mediator in steatohepatitis [9]. These data indicate that macrophage pyroptosis is mainly dependent on DDX3X-mediated NLRP3 activation in the pathogenesis of steatohepatitis.

### Macrophage DDX3X promotes M1 macrophage polarization, proinflammatory cytokines production, and hepatocyte steatosis

**Fig. 4.**
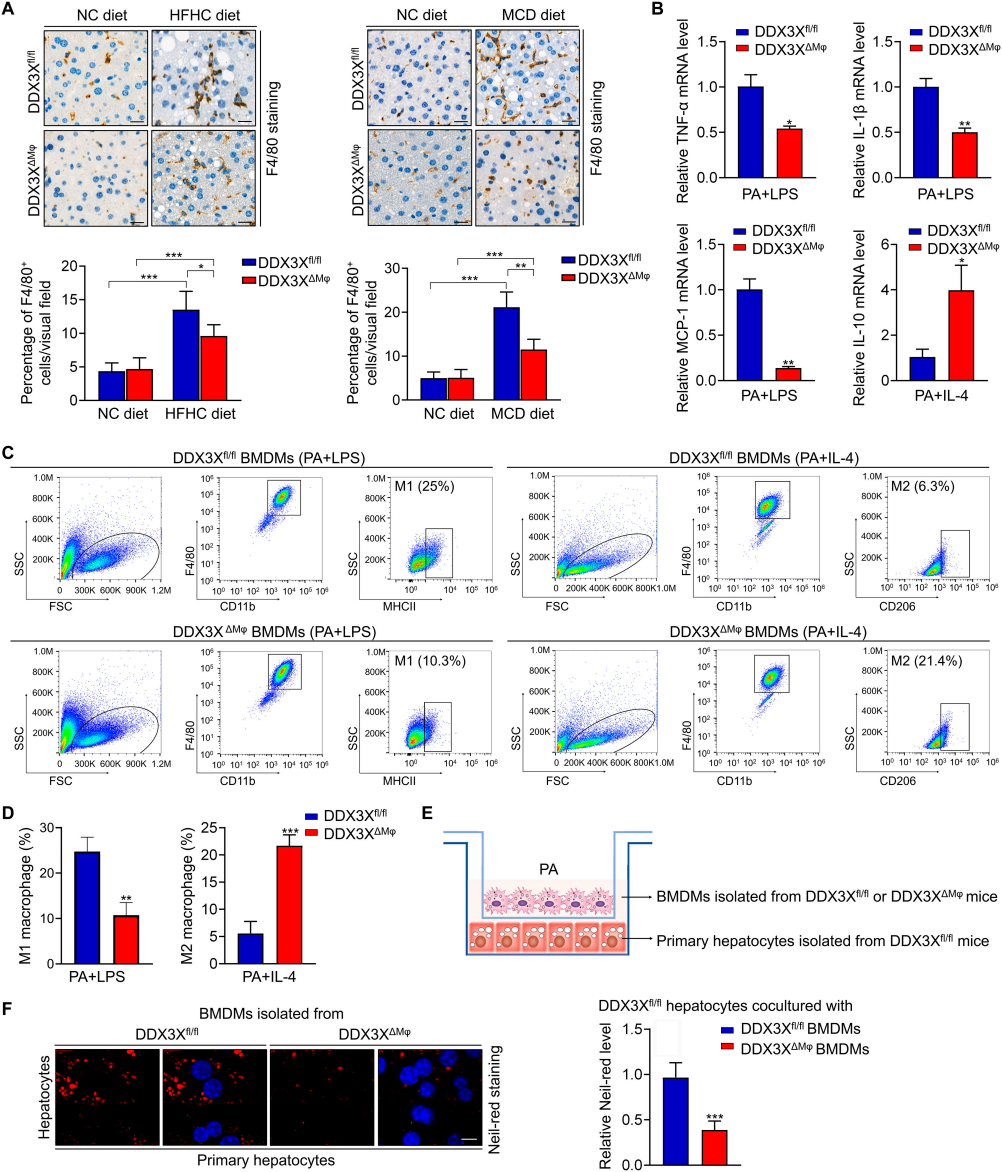
Macrophage DDX3X promotes M1 macrophage polarization, proinflammatory cytokines production, and hepatocyte steatosis. (A) Representative images of F4/80 staining in liver tissues of nutritional steatohepatitis. Scale bars, 20 μm. (B) mRNA expression of M1 and M2 macrophage markers in DDX3X^fl/fl^ and DDX3X^ΔMφ^ macrophages. (C and D) BMDMs isolated from DDX3X^fl/fl^ and DDX3X^ΔMφ^ mice were treated with PA + LPS or PA + IL-4 in vitro*.* Flow cytometry was used to detect M1 macrophages (F4/80^+^CD11b^+^MHCII^+^) and M2 macrophages (F4/80^+^CD11b^+^CD206^+^). (E) Schematic diagram of DDX3X^fl/fl^ or DDX3X^ΔMφ^ macrophages cocultured with DDX3X^fl/fl^ hepatocytes. (F) Representative Nile red-stained sections of DDX3X^fl/fl^ hepatocytes cocultured with DDX3X^fl/fl^ or DDX3X^ΔMφ^ macrophages. Scale bars, 10 μm. *n* = 3 to 6 per group. *P < 0.05, **P < 0.01, ***P < 0.001.

Macrophages are crucial innate immune cells involved in the regulation of hepatic pyroptosis and cell damage during the progression of NASH [9,24]. To explain the alleviated inflammatory response in the livers of DDX3X^ΔMφ^ mice, we assessed hepatic macrophage infiltration. F4/80 staining of liver sections revealed mitigated macrophage infiltration in DDX3X^ΔMφ^ mice compared to that in DDX3X^fl/fl^ mice (Fig. 4A). The NLRP3 signaling pathway was reported to be involved in the polarization of M1/M2 macrophages [25]. Loss of macrophage DDX3X inhibits NLRP3 activation. Therefore, we isolated BMDMs from DDX3X^fl/fl^ and DDX3X^ΔMφ^ mice and stimulated them in vitro with lipopolysaccharide (LPS) or IL-4 under conditions of PA induction to induce M1 or M2 macrophages. The mRNA expression of M1 macrophage markers [tumor necrosis factor-α (TNF-α), IL-1β, and MCP-1] was decreased in the DDX3X^ΔMφ^ BMDMs in comparison with the DDX3X^fl/fl^ BMDMs treated with PA + LPS (Fig. 4B). However, the mRNA expression levels of the M2 macrophage marker IL-10 were significantly increased in the DDX3X^ΔMφ^ BMDMs in comparison to the DDX3X^fl/fl^ BMDMs treated with PA + IL-4 (Fig. 4B). In addition, flow cytometry assays showed that DDX3X deletion in macrophages inhibited M1 macrophage polarization but promoted M2 macrophage polarization (Fig. 4C and D). To determine whether the amelioration of steatohepatitic changes found in DDX3X^ΔMφ^ mice was due to inhibiting NLRP3-mediated pyroptosis and decreasing M1 polarization, we cocultured DDX3X^fl/fl^ hepatocytes with DDX3X^fl/fl^ or DDX3X^ΔMφ^ macrophages in PA + LPS medium (Fig. 4E and F). Compared to DDX3X^fl/fl^ macrophages, DDX3X^ΔMφ^ macrophages cocultured with DDX3X^fl/fl^ hepatocytes showed significantly less lipid accumulation (Fig. 4F and Fig. S3).

### Hepatocyte DDX3X deletion exacerbates steatohepatitis in mice

**Fig. 5.**
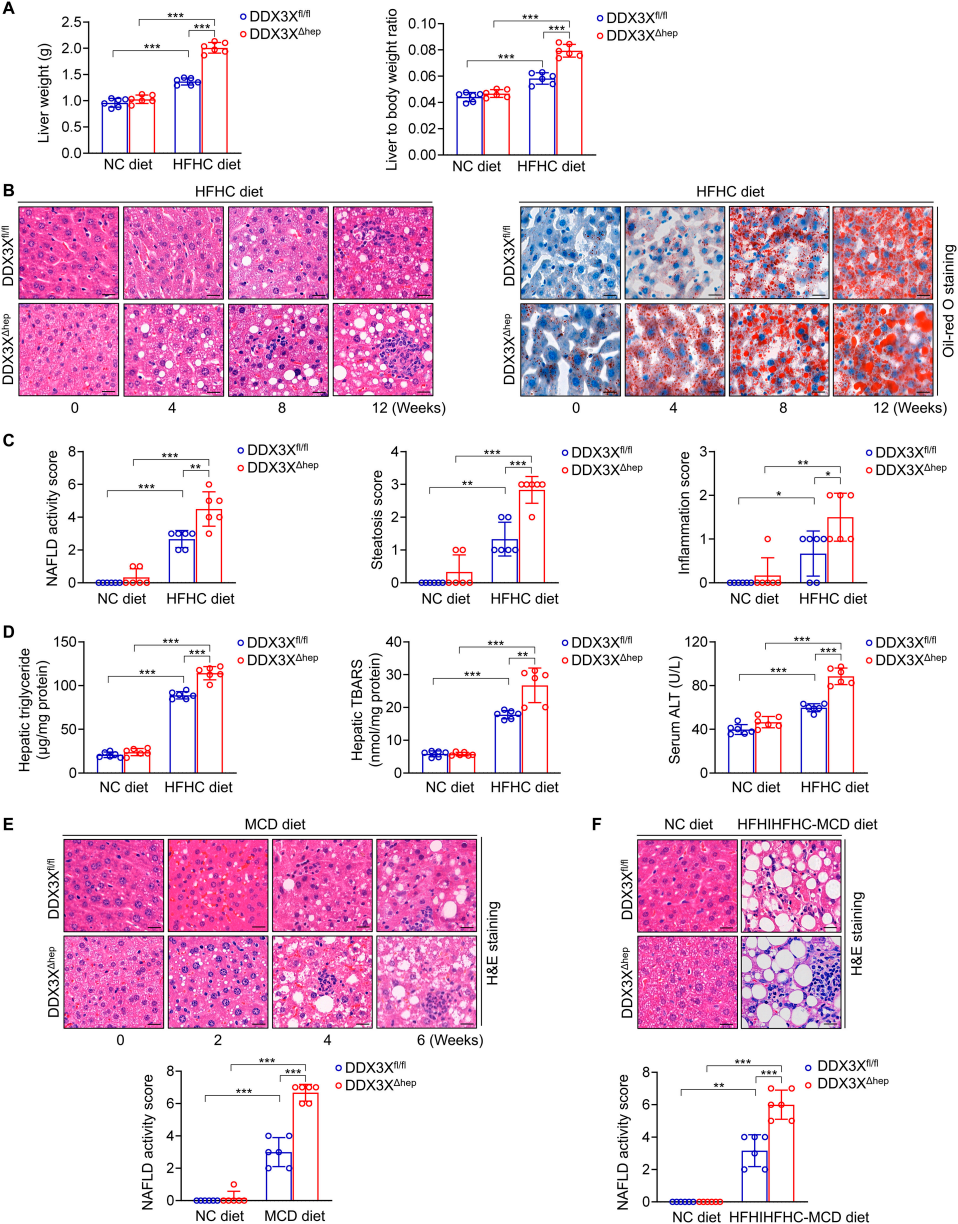
Deficiency of hepatocyte DDX3X exacerbates NASH progression. (A) Liver weights and liver-to-body weight ratios of DDX3X^fl/fl^ and DDX3X^Δhep^ mice exposed to the NC diet or HFHC diet for 12 weeks. (B) Histological evaluation of liver sections from DDX3X^fl/fl^ and DDX3X^Δhep^ mice fed an HFHC diet for 0, 4, 8, and 12 weeks. Scale bars, 20 μm. (C) NAFLD activity score, steatosis score, and inflammation score of liver sections were assessed in DDX3X^fl/fl^ and DDX3X^Δhep^ mice fed an HFHC diet for 12 weeks. (D) Hepatic TG content, liver lipid peroxidation, and serum ALT levels in DDX3X^fl/fl^ and DDX3X^Δhep^ mice fed an HFHC diet for 12 weeks. (E) Representative H&E-stained liver sections and NAFLD activity score of DDX3X^fl/fl^ and DDX3X^Δhep^ mice fed an MCD diet for 6 weeks. Scale bars, 20 μm. (F) Representative H&E-stained liver sections and NAFLD activity score of DDX3X^fl/fl^ and DDX3X^Δhep^ mice fed an HFHIHFHC-MCD diet for 6 weeks. Scale bars, 20 μm. *n* = 6 per group; data are expressed as the mean ± SD. *P < 0.05, **P < 0.01, ***P < 0.001.

Then, we explored the effect of hepatocyte DDX3X on the initiation and propagation of NASH by feeding DDX3X^Δhep^ mice and DDX3X^fl/fl^ control mice an HFHC diet for different durations (0, 4, 8, and 12 weeks). DDX3X was readily expressed in hepatocytes isolated from DDX3X^fl/fl^ mice but not in hepatocytes isolated from DDX3X^Δhep^ mice (Fig. S4A). DDX3X^Δhep^ mice showed higher liver weights and liver-to-body weight ratios than DDX3X^fl/fl^ mice fed the HFHC diet for 12 weeks (Fig. 5A). Importantly, histological examination of liver sections with different time courses of HFHC diet induction showed earlier and more notable lipid accumulation and inflammatory cell infiltration in DDX3X^Δhep^ mice than in DDX3X^fl/fl^ mice (Fig. 5B and C). Consistently, hepatic TG content, hepatic lipid peroxide levels, and serum ALT levels were all increased in DDX3X^Δhep^ mice compared to DDX3X^fl/fl^ mice fed the HFHC diet for 12 weeks (Fig. 5D), suggesting that hepatocyte DDX3X deletion promoted the progression of NASH. The effect of hepatocyte DDX3X on steatohepatitis was validated in two additional mouse models of steatohepatitis induced by MCD and HFHIHFHC-MCD diets. Similarly, DDX3X^Δhep^ mice showed more pronounced steatohepatitis in both models than DDX3X^fl/fl^ mice fed the same diet (Fig. 5E and F). Collectively, these data indicate that hepatocyte DDX3X deletion exacerbates steatohepatitis in mice.

### Hepatocyte DDX3X deletion impedes SG assembly in steatohepatitis

**Fig. 6.**
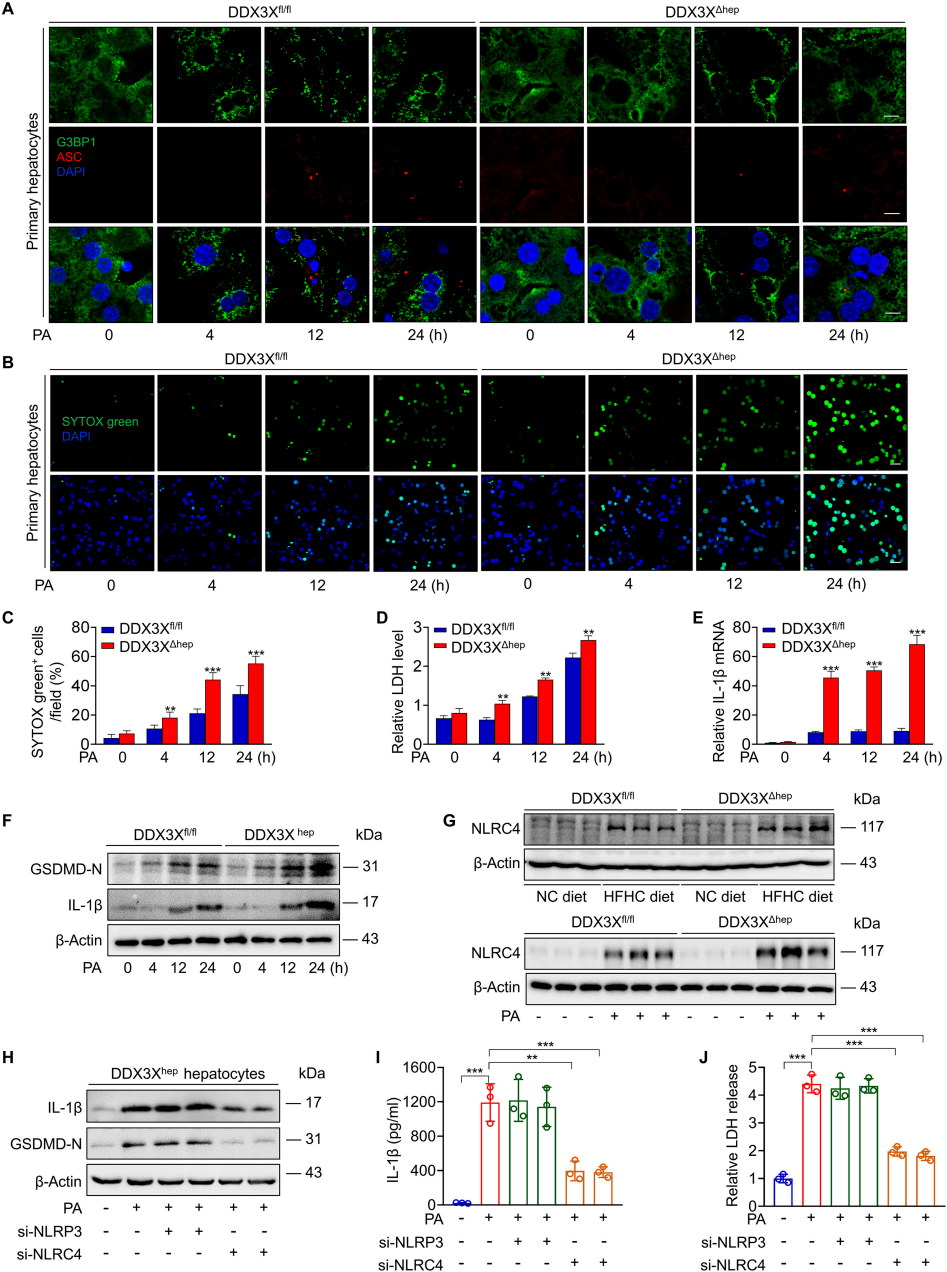
Hepatocyte DDX3X deletion causes defects in SG assembly and NLRP3 inflammasome activation but fails to protect against NLRC4-mediated hepatocyte pyroptosis. (A) Representative immunofluorescence images of G3BP1 and ASC in primary hepatocytes isolated from DDX3X^fl/fl^ or DDX3X^Δhep^ mice stimulated in vitro with PA (0.4 mM) for 0, 4, 12, and 24 h. Scale bars, 10 μm. (B and C) Representative immunofluorescence-stained sections of SYTOX green in primary hepatocytes isolated from DDX3X^fl/fl^ or DDX3X^Δhep^ mice with in vitro stimulation of PA (0.4 mM) for 0, 4, 12, and 24 h. Scale bars, 25 μm. (D) Medium LDH levels and (E) IL-1β expression of primary hepatocytes isolated from DDX3X^fl/fl^ or DDX3X^Δhep^ mice with in vitro stimulation of PA (0.4 mM) for 0, 4, 12, and 24 h, respectively. (F) GSDMD-N and IL-1β levels of primary hepatocytes isolated from DDX3X^fl/fl^ or DDX3X^Δhep^ mice stimulated with PA (0.4 mM) for 0, 4, 12, and 24 h. (G) NLRC4 protein levels in liver tissues or primary hepatocytes of DDX3X^fl/fl^ or DDX3X^Δhep^ mice in steatohepatitis. (H) GSDMD-N and IL-1β protein levels in PA-induced DDX3X^Δhep^ hepatocytes transfecting NLRP3 or NLRC4 small interfering RNA (siRNA). (I) IL-1β release and (J) medium LDH levels in PA-induced DDX3X^Δhep^ hepatocytes transfecting NLRP3 or NLRC4 siRNA. *n* = 3 per group; data are expressed as the mean ± SD. **P < 0.01, ***P < 0.001.

DDX3X is a central regulator between pro-survival SGs and the pyroptotic NLRP3 inflammasome under stress [10]. We then examined the role of DDX3X in hepatocyte SG assembly and NLRP3 activation in NASH. Interestingly, both hepatocytes and BMDMs showed G3BP1 distribution after PA induction, but hepatocytes exhibited higher G3BP1 protein levels than BMDMs isolated from the same mice (Fig. S4B). The expression of NLRP3 inflammasome components was significantly higher in macrophages than in hepatocytes after PA induction (Fig. S4B). Co-IP experiments showed that DDX3X could interact with both G3BP1 and NLRP3 under PA induction in primary hepatocytes (Fig. S4C). Confocal microscopy imaging of G3BP1 in DDX3X^fl/fl^ hepatocytes showed obvious assembly of numerous SGs as early as 4 h after PA induction (Fig. 6A). The number of assembled SGs peaked for approximately 12 h after PA induction, and disassembly gradually occurred over time (Fig. 6A). However, compared to DDX3X^fl/fl^ hepatocytes, DDX3X^Δhep^ hepatocytes exhibited impaired SG assembly (Fig. 6A), indicating that severe steatohepatitis caused by hepatocyte DDX3X deletion may at least partly result from the inhibition of pro-survival SG formation, thus impairing the ability of hepatocytes to respond to changes in homeostatic flux. To further address the role of SGs in steatohepatitis, we knocked down hepatocyte G3BP1 in mice by injecting AAV8-*mi*G3BP1 containing the hepatocyte-specific promoter TBG, and the mice were fed an HFHC diet for 12 weeks. G3BP1 was specifically knocked down in hepatocytes (Fig. S5A to C). Mice lacking G3BP1 in hepatocytes had more severe steatosis and a more aggravated steatohepatitis phenotype than control mice (Fig. S5D and E), further validating that hepatocyte SGs protected mice from steatohepatitis.

### Hepatocyte DDX3X deletion suppresses NLRP3 inflammasome activation but fails to protect against NLRC4-mediated hepatocyte pyroptosis

Then, we evaluated NLRP3 inflammasome activation in hepatocytes. Similar to macrophages, the appearance of pyroptotic ASC specks was a relatively late event during PA treatment compared to the formation of SGs. DDX3X^fl/fl^ hepatocytes showed pyroptotic ASC specks after approximately 12 h of PA treatment and were progressively enhanced with PA insult thereafter (Fig. 6A). Pyroptotic ASC specks were relatively decreased in hepatocytes isolated from DDX3X^Δhep^ mice (Fig. 6A). However, monitoring of cell death by SYTOX Green dye staining demonstrated that DDX3X^Δhep^ hepatocytes were more sensitive to the pro-death phenotype after PA treatment than DDX3X^fl/fl^ hepatocytes (Fig. 6B and C). More importantly, both LDH and IL-1β production were substantially enhanced in DDX3X^Δhep^ hepatocytes compared with DDX3X^fl/fl^ hepatocytes (Fig. 6D to F). Consistently, DDX3X^Δhep^ hepatocytes showed more obvious GSDMD cleavage than DDX3X^fl/fl^ hepatocytes with PA induction (Fig. 6F), indicating that hepatocyte pyroptosis was not completely dependent on DDX3X-mediated NLRP3 activation. These results are consistent with a previous study demonstrating that in contrast to NLRP3-dominant macrophage pyroptosis, NLRC4-mediated caspase-1 activation, but not NLRP3, is indispensable for hepatocyte pyroptosis in HFHC diet-fed steatohepatitis [26]. Consistently, examination of the expression and distribution of NLRC4 in primary hepatocytes and BMDMs also confirmed that NLRC4 was predominantly expressed in primary hepatocytes under PA conditions (Fig. S4B). Significant up-regulation of NLRC4 expression in DDX3X^Δhep^ liver tissues and primary hepatocytes in steatohepatitis was further confirmed both in vivo and in vitro (Fig. 6G). In order to further verify the role of DDX3X in the SGs, NLRP3, and NLRC4 inflammasome in hepatocytes, we applied the specific activators of these protein machines in DDX3X^Δhep^ hepatocytes. As shown in Fig. S6A, applying arsenite, an inducer of SGs, failed to induce the assembly of SGs in DDX3X^Δhep^ hepatocytes, indicating that DDX3X was essential in hepatocyte SG formation. In addition, compared with DDX3X^fl/fl^ hepatocytes, using the NLRP3 inflammasome activator nigericin in DDX3X^Δhep^ hepatocytes failed to induce NLRP3 inflammasome activation (Fig. S6B to E), while adding the NLRC4 inflammasome activator flagellin significantly induced pyroptosis of DDX3X^Δhep^ hepatocytes (Fig. S6F to H), suggesting that hepatocyte NLRP3 inflammasome activation was DDX3X dependent, while hepatocyte NLRC4 inflammasome activation was DDX3X independent, consistent with a previous study in BMDMs [10]. Moreover, knocking down NLRP3 did not inhibit PA-induced DDX3X^Δhep^ hepatocyte pyroptosis, while silencing NLRC4 significantly alleviated pyroptosis in PA-induced DDX3X^Δhep^ hepatocytes, further proving that PA-induced pyroptosis in DDX3X^Δhep^ hepatocytes was mainly caused by NLRC4 inflammasome activation rather than the NLRP3 inflammasome (Fig. 6H to J). All the findings indicate that loss of SG protection caused by hepatocyte DDX3X deficiency, as well as pro-pyroptotic NLRC4 activation, coordinately accelerate steatohepatitis.

### Loss of DDX3X in hepatocytes promotes macrophage infiltration and M1 macrophage polarization

**Fig. 7.**
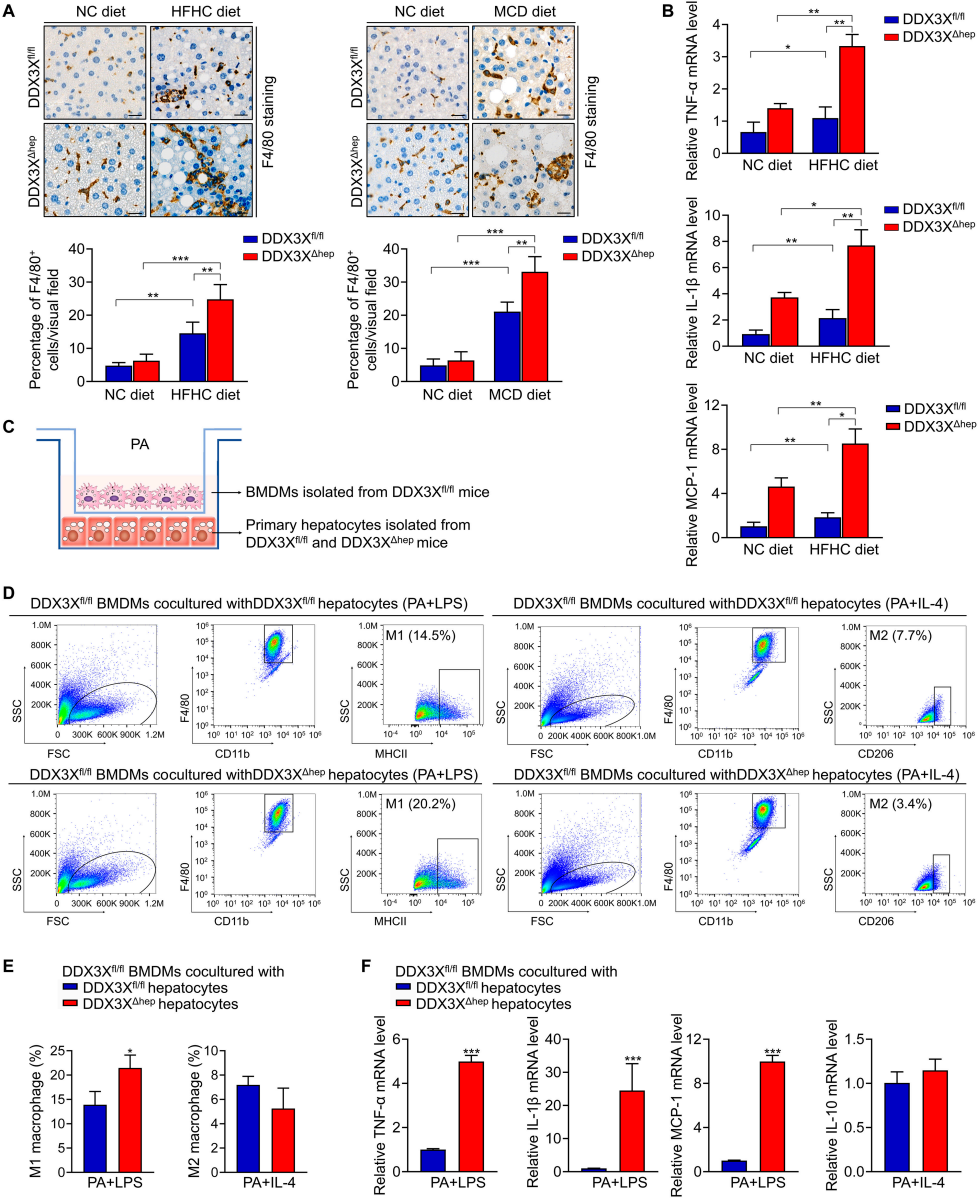
Hepatocyte DDX3X deficiency induces macrophage infiltration and M1 macrophage polarization. (A) Representative F4/80-stained sections in liver tissues of nutritional steatohepatitis. Scale bars, 20 μm. (B) Hepatic TNF-α, IL-1β, and MCP-1 mRNA expression levels in liver tissues of DDX3X^fl/fl^ or DDX3X^Δhep^ mice. (C) Schematic diagram of DDX3X^fl/fl^ macrophages cocultured with DDX3X^fl/fl^ or DDX3X^Δhep^ hepatocytes. (D and E) F4/80^+^CD11b^+^MHCII^+^ M1 macrophages and F4/80^+^CD11b^+^CD206^+^ M2 macrophages in BMDMs isolated from DDX3X^fl/fl^ mice and then cocultured with primary hepatocytes isolated from DDX3X^fl/fl^ or DDX3X^Δhep^ mice treated with PA + LPS or PA + IL-4 by flow cytometry. (F) mRNA expression of TNF-α, IL-1β, and MCP-1 (M1 macrophage markers) and IL-10 (M2 macrophage marker) in DDX3X^fl/fl^ macrophages when cocultured with DDX3X^fl/fl^ or DDX3X^Δhep^ hepatocytes under PA conditions. *n* = 3 to 6 per group; data are expressed as the mean ± SD. *P < 0.05, **P < 0.01, ***P < 0.001.

To explain the marked inflammatory response in the livers of DDX3X^Δhep^ mice, we performed F4/80 immunostaining to evaluate macrophage infiltration. Immunohistochemical (IHC) staining of liver sections revealed more macrophage infiltration in DDX3X^Δhep^ mice than in DDX3X^fl/fl^ mice exposed to an HFHC diet or an MCD diet (Fig. 7A). In an in vitro coculture model, macrophages isolated from DDX3X^fl/fl^ mice cocultured with DDX3X^Δhep^ hepatocytes also showed enhanced infiltration ability compared to those cocultured with DDX3X^fl/fl^ hepatocytes (Fig. S6I). Subsequently, we analyzed pyroptosis-related proinflammatory cytokines mRNA expression levels in the liver tissues of DDX3X^fl/fl^ and DDX3X^Δhep^ mice. Hepatic TNF-α, IL-1β, and MCP-1 mRNA expression was increased in the liver tissues of DDX3X^Δhep^ mice compared to those of DDX3X^fl/fl^ mice fed the HFHC diet (Fig. 7B), suggesting that loss of DDX3X in hepatocytes promoted proinflammatory cytokines production. Given that up-regulation of these proinflammatory cytokines was tightly associated with M1 macrophage polarization, we analyzed M1/M2 macrophage phenotypes. Indeed, after PA + LPS induction, DDX3X^fl/fl^ macrophages cocultured with DDX3X^Δhep^ hepatocytes showed enhanced M1 macrophage polarization compared to DDX3X^fl/fl^ macrophages cocultured with DDX3X^fl/fl^ hepatocytes in vitro (Fig. 7C to E). Consistently, the mRNA expression levels of M1 macrophage markers were considerably higher in DDX3X^fl/fl^ macrophages cocultured with DDX3X^Δhep^ hepatocytes. However, IL-10 (M2 macrophage marker) mRNA expression was not changed significantly in DDX3X^fl/fl^ macrophages cocultured with DDX3X^Δhep^ hepatocytes (Fig. 7F). These data suggest that loss of DDX3X in hepatocytes induces macrophage infiltration and M1 macrophage polarization, thus facilitating inflammatory responses in steatohepatitis.

## Discussion

**Fig. 8.**
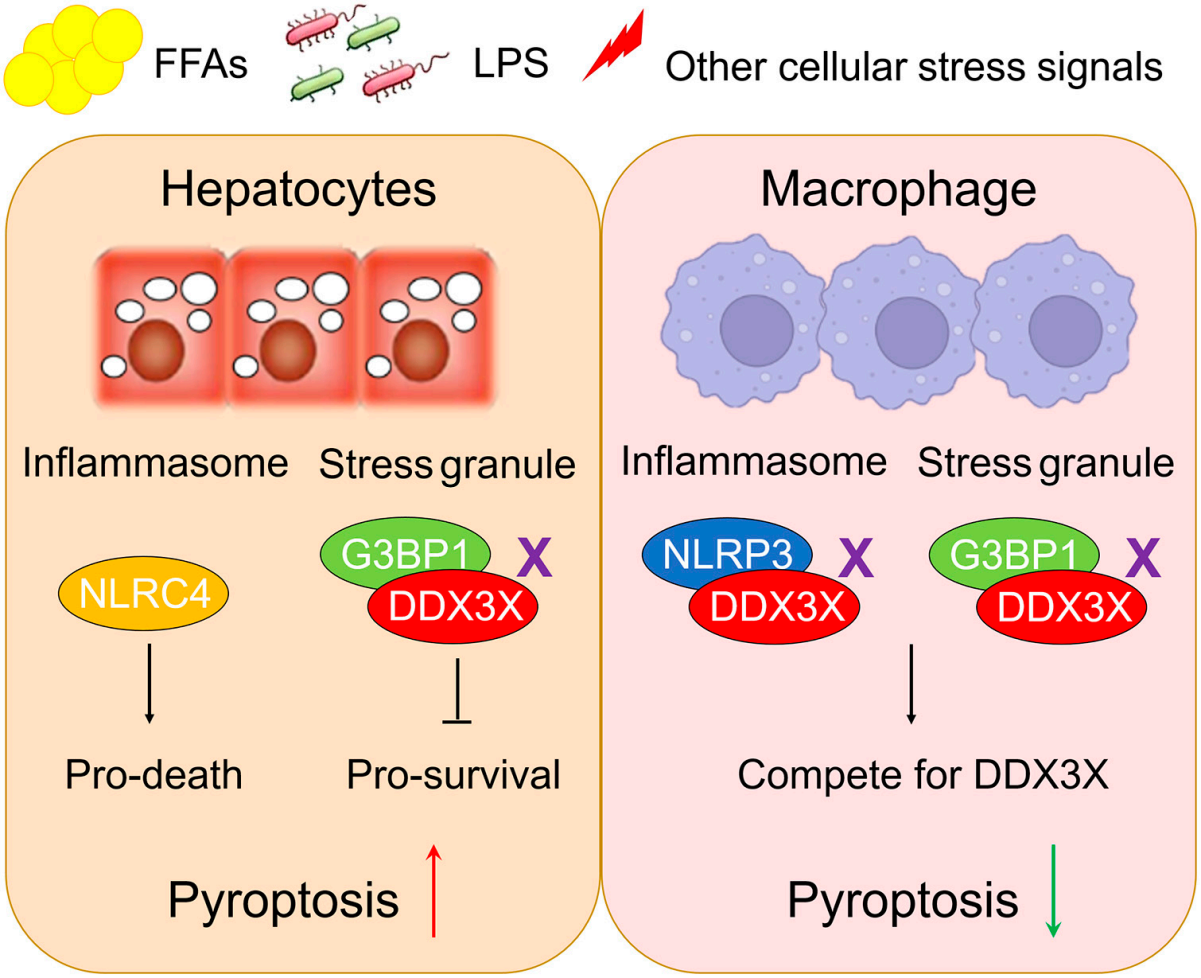
Schematic diagram of the role of DDX3X in nonalcoholic steatohepatitis. Macrophage DDX3X deletion inhibits NLRP3-mediated pyroptosis and improves liver histology in nutritional steatohepatitis. This action is mainly due to the decisive role of NLRP3 in controlling inflammation and pyroptosis in macrophages. However, DDX3X deletion in hepatocytes significantly exacerbates histological phenotypes in multiple nutritional steatohepatitis models. Hepatocyte DDX3X deletion impairs SG assembly, leading to increased sensitivity and intolerance to PA stimulation and resultant steatohepatitis. Although hepatocyte DDX3X deletion inhibits NLRP3 inflammasome activation spontaneously, DDX3X does not affect the extensive activation of NLRC4, the major inflammasome that induces hepatocyte pyroptosis in steatohepatitis. Therefore, loss of SG protection caused by hepatocyte DDX3X deficiency, as well as pro-pyroptotic NLRC4 activation, coordinately accelerates steatohepatitis.

The study reveals that DDX3X plays a vital role in the progression of NASH (Fig. 8). DDX3X was found to be predominantly expressed in macrophages and hepatocytes in the liver tissues of humans and mice, and DDX3X expression was decreased with NASH. In hepatocytes and macrophages, DDX3X played opposing roles in the progression of NASH. Macrophage DDX3X deletion inhibited NLRP3-mediated pyroptosis and improved liver histology in nutritional steatohepatitis. Hepatocyte DDX3X deletion impaired SG assembly, leading to increased sensitivity and intolerance to metabolic stimulation and NLRC4 inflammasome activation, coordinately exacerbating steatohepatitis and resultant steatohepatitis.

DDX3X is a central decision marker governing the crosstalk between pro-survival SGs or pro-pyroptotic NLRP3 inflammasomes formed in stressed cells, thus controlling whether cells live or die [10,11]. Our previous work demonstrated that GSDMD-driven pyroptosis acted as a critical inflammatory mediator in NASH [9]. Persistent hepatic cellular metabolic stress and liver inflammatory stimuli are key signatures of NASH, suggesting that DDX3X may play a crucial role in NASH progression. Previous investigation has shown that DDX3X reduces steatosis during NAFLD progression [7]. However, the role of DDX3X in inflammation is not fully explored, and the function of DDX3X in NASH remains unclear. In particular, the cell-specific function of DDX3X and underlying mechanisms are still unknown. Therefore, we explored the function of hepatocyte and macrophage DDX3X in the progression of NASH. Given that NLRP3 in myeloid cells exerts a crucial function in experimental steatohepatitis [23] and that decreased DDX3X expression was also found in macrophages in steatohepatitis, we evaluated the role of DDX3X in governing the crosstalk between pro-survival and pro-death cell fate pathways in BMDMs and found that macrophage DDX3X deletion improved liver histology in multiple nutritional steatohepatitis (Fig. 2). Both SG assembly and NLRP3 activation were impaired in DDX3X^ΔMφ^ macrophages compared to DDX3X^fl/fl^ macrophages treated with PA (Fig. 3). Mechanistically, the involvement of macrophage DDX3X is mainly dependent on DDX3X-mediated NLRP3 activation and M1 macrophage polarization, that is, crosstalk with hepatocytes to induce hepatocyte steatohepatitic changes and steatohepatitis development. In contrast, DDX3X^Δhep^ mice displayed more severe steatohepatitis with pronounced liver injury, lipid accumulation, and inflammatory cell infiltration than DDX3X^fl/fl^ mice in multiple nutritional steatohepatitis models (Fig. 5). Further investigation revealed that although SG assembly and NLRP3 activation coexisted in steatohepatitis, SG formation was a relatively early event in the development of steatohepatitis, and hepatocyte DDX3X deletion significantly inhibited SG assembly, thus failing to allow the cell to persist. Consequently, earlier and more notable pyroptotic cell death was found in hepatocytes of DDX3X^Δhep^ mice compared to DDX3X^fl/fl^ mice with PA induction. NLRP3 inflammasome activation was a relatively late event in the development of steatohepatitis compared to SG assembly. After long-term PA induction, pyroptotic ASC specks were also defective in hepatocytes that were deficient in DDX3X. However, DDX3X^Δhep^ hepatocytes were still more sensitive to the pro-death phenotype than DDX3X^fl/fl^ hepatocytes, indicating that hepatocyte pyroptosis was not completely dependent on DDX3X-mediated NLRP3 activation. A previous study demonstrated that NLRC4-dependent pyroptosis in hepatocytes was required for the development of NASH [26]. We wondered whether the pro-pyroptotic phenotype found in DDX3X^Δhep^ hepatocytes was partly due to NLRC4 inflammasome activation. To confirm this speculation, we analyzed the expression of NLRC4, NLRP3, and the adaptor protein ASC and their distribution in hepatocytes and BMDMs. The different distribution of NLRP3 and NLRC4 in BMDMs and hepatocytes may be one factor contributing to the determination of the dominant activated inflammasome type. All these findings demonstrated that hepatocytes inevitably succumbed to pyroptosis due to NLRC4 inflammasome activation, even when NLRP3 inflammasome activation was blocked by DDX3X deletion. Hepatic pyroptosis controls IL-1β production, which has been reported to promote the production of other proinflammatory cytokines (such as TNF-α and MCP-1) and aggravate the inflammatory response in liver disease [9,27,28]. Consistently, hepatic IL-1β, TNF-α, and MCP-1 mRNA expression levels were all dramatically increased in the liver tissues of DDX3X^Δhep^ mice in comparison with DDX3X^fl/fl^ mice with nutritional steatohepatitis, suggesting that loss of DDX3X in hepatocytes promoted proinflammatory cytokine production. Given that up-regulation of these proinflammatory cytokines is tightly associated with M1 macrophage polarization, we analyzed M1/M2 macrophage phenotypes. We found enhanced macrophage infiltration and M1 macrophage polarization when DDX3X^fl/fl^ macrophages were cocultured with DDX3X^Δhep^ hepatocytes compared to DDX3X^fl/fl^ macrophages cocultured with DDX3X^fl/fl^ hepatocytes. These results indicate that hepatocyte DDX3X deficiency induces steatohepatitis, at least partly through crosstalk with macrophages and the promotion of M1 macrophage infiltration.

Collectively, this work identified a cell-specific role of DDX3X in NASH progression. Macrophage DDX3X deletion inhibited NLRP3-mediated pyroptosis and improved liver histology in nutritional steatohepatitis. However, hepatocyte DDX3X deletion mainly caused defects in SG assembly, leading to increased sensitivity and intolerance to metabolic stimulation and NLRC4 inflammasome activation, exacerbating steatohepatitis. It would be worthwhile to understand whether cell-specific differences exist in the crosstalk between SG formation and NLRP3 inflammasome activation for future clinical trials and to develop targeted DDX3X drugs for the treatment of NASH.

## Materials and Methods

### Human samples

Human NASH liver tissues (*n* = 14) were obtained from patients undergoing bariatric surgery without liver diseases resulting from other etiologies from the Affiliated Jinling Hospital of Nanjing University Medical School. Control liver samples (*n *= 10) originated from patients with hepatic hemangioma. Histological assessments were performed in a double-blind manner by two pathologists [29,30]. Written informed consent was obtained from all patients, and all procedures were conducted under the supervision of the Clinical Research Ethics Committee of Nanjing University, which is in compliance with the ethical guidelines of the 1975 Declaration of Helsinki.

### Mice and treatments

The DDX3X floxed allele was generated in mice on a C57BL/6 background using CRISPR–Cas9 gene editing as previously described [10]. The floxed allele was bred into homozygotes to generate DDX3X^fl/fl^ mice. DDX3X^Δhep^ mice were generated by crossing DDX3X^fl/fl^ mice with Alb-Cre mice (C57BL/6 strain). DDX3X^ΔMφ^ mice were generated by crossing DDX3X^fl/fl^ mice with Lyz2-Cre mice (C57BL/6 strain). DDX3X^fl/fl^ littermates were used as respective wild-type controls.

Male DDX3X^Δhep^ mice, DDX3X^ΔMφ^ mice, and DDX3X^fl/fl^ littermates (8 weeks old, 6 per group) were randomly fed an NC diet or a high-fat/high-cholesterol (HFHC) diet (fat: 40% kcal with 1.25% cholesterol) and sacrificed at 0, 4, 8, and 12 weeks after induction. In a separate experiment, male DDX3X^Δhep^ mice, DDX3X^ΔMφ^ mice, and DDX3X^fl/fl^ littermates (8 weeks old, 6 mice per group) were randomly fed an NC diet or an MCD diet and sacrificed at 0, 2, 4, and 6 weeks after induction. In addition, male DDX3X^Δhep^ mice, DDX3X^ΔMφ^ mice, and DDX3X^fl/fl^ littermates (8 weeks old, 6 mice per group) were randomly fed an HFHIHFHC-MCD diet with 40 kcal% fat, high iron (100 parts per million), high fructose (25%), high cholesterol (1%), low methionine (0.12%), and no added choline for 6 weeks to generate another nutritional steatohepatitis model. Mice were fasted overnight before sacrifice, and liver tissues and serum were collected for further evaluation as previously reported [2]. All animal experiments met the standards of the Institutional Animal Use and the Animal Experimentation Ethics Committee at the Northwest University of China.

## Data Availability

Data are available upon request from the corresponding author.

## Supplementary Materials

Supplementary 1 Supplementary Materials and Methods Figs. S1 to S6 Table S1 Western blot data

## References

[1] Wang Q, Zhou H, Bu Q, Wei S, Li L, Zhou J, Zhou S, Su W, Liu M, Liu Z, et al. Role of XBP1 in regulating the progression of non-alcoholic steatohepatitis. J Hepatol. 2022;77(2):312–325. 35292349 10.1016/j.jhep.2022.02.031

[2] Zhang X, Fan L, Wu J, Xu H, Leung WY, Fu K, Wu J, Liu K, Man K, Yang X, et al. Macrophage p38α promotes nutritional steatohepatitis through M1 polarization. J Hepatol. 2019;71(1):163–174. 30914267 10.1016/j.jhep.2019.03.014

[3] Zhang Z, Xu X, Tian W, Jiang R, Lu Y, Sun Q, Fu R, He Q, Wang J, Liu Y, et al. ARRB1 inhibits non-alcoholic steatohepatitis progression by promoting GDF15 maturation. J Hepatol. 2020;72(5):976–989. 31857195 10.1016/j.jhep.2019.12.004

[4] Schuster S, Cabrera D, Arrese M, Feldstein AE. Triggering and resolution of inflammation in NASH. Nat Rev Gastroenterol Hepatol. 2018;15(6):349–364. 29740166 10.1038/s41575-018-0009-6

[5] Wong VW, Chitturi S, Wong GL, Yu J, Chan HL, Farrell GC. Pathogenesis and novel treatment options for non-alcoholic steatohepatitis. Lancet Gastroenterol Hepatol. 2016;1(1):56–67. 28404113 10.1016/S2468-1253(16)30011-5

[6] Tsai TY, Wang WT, Li HK, Chen WJ, Tsai YH, Chao CH, Wu Lee YH. RNA helicase DDX3 maintains lipid homeostasis through upregulation of the microsomal triglyceride transfer protein by interacting with HNF4 and SHP. Sci Rep. 2017;7:41452. 28128295 10.1038/srep41452 PMC5269733

[7] Liu P, Zhang Y, Tang C, Cen L, Chen Y, Li S, Chen X, Yu M, Zhang J, Zhang X, et al. The DEAD-box helicase DDX3x ameliorates non-alcoholic fatty liver disease via mTORC1 signalling pathway. Liver Int. 2022;42(8):1793–1802. 35460172 10.1111/liv.15278

[8] Loomba R, Friedman SL, Shulman GI. Mechanisms and disease consequences of nonalcoholic fatty liver disease. Cell. 2021;184(10):2537–2564. 33989548 10.1016/j.cell.2021.04.015 PMC12168897

[9] Xu B, Jiang M, Chu Y, Wang W, Chen D, Li X, Zhang Z, Zhang D, Fan D, Nie Y, et al. Gasdermin D plays a key role as a pyroptosis executor of non-alcoholic steatohepatitis in humans and mice. J Hepatol. 2018;68(4):773–782. 29273476 10.1016/j.jhep.2017.11.040

[10] Samir P, Kesavardhana S, Patmore DM, Gingras S, Malireddi RKS, Karki R, Guy CS, Briard B, Place DE, Bhattacharya A, et al. DDX3X acts as a live-or-die checkpoint in stressed cells by regulating NLRP3 inflammasome. Nature. 2019;573(7775):590–594. 31511697 10.1038/s41586-019-1551-2 PMC6980284

[11] Fox D, Man SM. DDX3X: Stressing the NLRP3 inflammasome. Cell Res. 2019;29(12):969–970. 31659248 10.1038/s41422-019-0250-8 PMC6951334

[12] Wang S, Kwon SH, Su Y, Dong Z. Stress granules are formed in renal proximal tubular cells during metabolic stress and ischemic injury for cell survival. Am J Physiol Renal Physiol. 2019;317(1):F116–F123. 31091124 10.1152/ajprenal.00139.2019 PMC6692727

[13] Wen WL, Stevenson AL, Wang CY, Chen HJ, Kearsey SE, Norbury CJ, Watt S, Bahler J, Wang SW. Vgl1, a multi-KH domain protein, is a novel component of the fission yeast stress granules required for cell survival under thermal stress. Nucleic Acids Res. 2010;38(19):6555–6566. 20547592 10.1093/nar/gkq555 PMC2965253

[14] Xue Y, Enosi Tuipulotu D, Tan WH, Kay C, Man SM. Emerging activators and regulators of inflammasomes and pyroptosis. Trends Immunol. 2019;40(11):1035–1052. 31662274 10.1016/j.it.2019.09.005

[15] Man SM, Karki R, Kanneganti TD. Molecular mechanisms and functions of pyroptosis, inflammatory caspases and inflammasomes in infectious diseases. Immunol Rev. 2017;277(1):61–75. 28462526 10.1111/imr.12534 PMC5416822

[16] Shi J, Zhao Y, Wang K, Shi X, Wang Y, Huang H, Zhuang Y, Cai T, Wang F, Shao F. Cleavage of GSDMD by inflammatory caspases determines pyroptotic cell death. Nature. 2015;526(7575):660–665. 26375003 10.1038/nature15514

[17] Jorgensen I, Miao EA. Pyroptotic cell death defends against intracellular pathogens. Immunol Rev. 2015;265(1):130–142. 25879289 10.1111/imr.12287 PMC4400865

[18] Fink SL, Cookson BT. Caspase-1-dependent pore formation during pyroptosis leads to osmotic lysis of infected host macrophages. Cell Microbiol. 2006;8(11):1812–1825. 16824040 10.1111/j.1462-5822.2006.00751.x

[19] Shi J, Gao W, Shao F. Pyroptosis: Gasdermin-mediated programmed necrotic cell death. Trends Biochem Sci. 2017;42(4):245–254. 27932073 10.1016/j.tibs.2016.10.004

[20] Kadono K, Kageyama S, Nakamura K, Hirao H, Ito T, Kojima H, Dery KJ, Li X, Kupiec-Weglinski JW. Myeloid Ikaros-SIRT1 signaling axis regulates hepatic inflammation and pyroptosis in ischemia-stressed mouse and human liver. J Hepatol. 2022;76(4):896–909. 34871625 10.1016/j.jhep.2021.11.026 PMC9704689

[21] Khanova E, Wu R, Wang W, Yan R, Chen Y, French SW, Llorente C, Pan SQ, Yang Q, Li Y, et al. Pyroptosis by caspase11/4-gasdermin-D pathway in alcoholic hepatitis in mice and patients. Hepatology. 2018;67(5):1737–1753. 29108122 10.1002/hep.29645 PMC5906140

[22] Gaul S, Leszczynska A, Alegre F, Kaufmann B, Johnson CD, Adams LA, Wree A, Damm G, Seehofer D, Calvente CJ, et al. Hepatocyte pyroptosis and release of inflammasome particles induce stellate cell activation and liver fibrosis. J Hepatol. 2021;74(1):156–167. 32763266 10.1016/j.jhep.2020.07.041 PMC7749849

[23] Kaufmann B, Kui L, Reca A, Leszczynska A, Kim AD, Booshehri LM, et al. Cell-specific deletion of NLRP3 inflammasome identifies myeloid cells as key drivers of liver inflammation and fibrosis in murine steatohepatitis. Cell Mol Gastroenterol Hepatol. 2022;14:751–767. 35787975 10.1016/j.jcmgh.2022.06.007 PMC9424559

[24] Kazankov K, Jørgensen SMD, Thomsen KL, Møller HJ, Vilstrup H, George J, Schuppan D, Grønbæk H. The role of macrophages in nonalcoholic fatty liver disease and nonalcoholic steatohepatitis. Nat Rev Gastroenterol Hepatol. 2019;16(3):145–159. 30482910 10.1038/s41575-018-0082-x

[25] Li C, Jin Y, Wei S, Sun Y, Jiang L, Zhu Q, Farmer DG, Busuttil RW, Kupiec-Weglinski JW, Ke B. Hippo signaling controls NLR family pyrin domain containing 3 activation and governs immunoregulation of mesenchymal stem cells in mouse liver injury. Hepatology. 2019;70(5):1714–1731. 31063235 10.1002/hep.30700 PMC6819196

[26] Koh EH, Yoon JE, Ko MS, Leem J, Yun JY, Hong CH, Cho YK, Lee SE, Jang JE, Baek JY, et al. Sphingomyelin synthase 1 mediates hepatocyte pyroptosis to trigger non-alcoholic steatohepatitis. Gut. 2021;70(10):1954–1964. 33208407 10.1136/gutjnl-2020-322509 PMC8458090

[27] Petrasek J, Bala S, Csak T, Lippai D, Kodys K, Menashy V, Barrieau M, Min SY, Kurt-Jones EA, Szabo G. IL-1 receptor antagonist ameliorates inflammasome-dependent alcoholic steatohepatitis in mice. J Clin Invest. 2012;122(10):3476–3489. 22945633 10.1172/JCI60777 PMC3461900

[28] Petrasek J, Dolganiuc A, Csak T, Kurt-Jones EA, Szabo G. Type I interferons protect from Toll-like receptor 9-associated liver injury and regulate IL-1 receptor antagonist in mice. Gastroenterology. 2011;140(2):697–708.e4. 20727895 10.1053/j.gastro.2010.08.020 PMC3031737

[29] Sanyal AJ, Brunt EM, Kleiner DE, Kowdley KV, Chalasani N, Lavine JE, Ratziu V, McCullough A. Endpoints and clinical trial design for nonalcoholic steatohepatitis. Hepatology. 2011;54(1):344–353. 21520200 10.1002/hep.24376 PMC4014460

[30] Kleiner DE, Brunt EM, Van Natta M, Behling C, Contos MJ, Cummings OW, et al. Design and validation of a histological scoring system for nonalcoholic fatty liver disease. Hepatology. 2005;41(6):1313–1321. 15915461 10.1002/hep.20701

