# Supplementary material for: Protective and Adverse Roles of DDX3X in Different Cell Types in Nonalcoholic Steatohepatitis Progression
Source: Research (Wash D C). 2023 Dec 8;6:0275. doi: 10.34133/research.0275 (PMC10712874; doi:10.34133/research.0275)
Supplement: Supplementary 1 — Supplementary Materials and Methods Figs. S1 to S6 Table S1 Western blot data [file research.0275.f1.docx]

Protective and adverse roles of DDX3X in different cell types in non-alcoholic steatohepatitis progression

Suzhen Yang^1, #^, Lin Zhou^1, #^, Tianming Zhao^1^^, #^, Hanlong Zhu^2, #^, Tingting Luo^3^, Kang Jiang^2^, Xiaoxiao Shi^2^, Chunyan Chen^2^, Han Zhang^1^, Si Zhao^1^, Xiaoping Zou^1^, Yuzheng Zhuge^1,^*, Fangyu Wang^2,^*, Lei Wang^1,^*, Mingzuo Jiang^2,^*, Bing Xu^1,^*

**Table of contents**

Supplementary Materials and Methods ........................................................ 1

Figure. S1 ..................................................................................................... 6

Figure. S2 ..................................................................................................... 7

Figure. S3 ..................................................................................................... 7

Figure. S4 ..................................................................................................... 8

Figure. S5 ..................................................................................................... 9

Figure. S6 ..................................................................................................... 10

Table S1 ....................................................................................................... 11

Western bolts data ........................................................................................12

**Supplementary materials and methods**

**Cell isolation, cell culture and co-culture**

Primary hepatocytes were isolated from DDX3X^fl/fl^ and DDX3X^Δhep^ mice. 40 ml of 37°C pre-heated Krebs-Ringer buffer (Solarbio, Beijing, China) was pumped from the portal vein of the mice, followed by 20 ml of 37°C pre-warmed 0.3% type IV collagenase (Worthington Biochemical, Lakewood, USA) for digestion. Then the liver was gently removed and placed in pre-chilled DMEM medium to isolate the cells. The cell suspension was passed through a 70µm cell strainer (Corning, Arkansas, USA). After centrifugation at 50g for 5 minutes, the cell precipitate was obtained as hepatocytes. The isolated hepatocytes were seeded in cell culture plate previously coated with Rat Tail Collagen Type I (Corning, Arkansas, USA) and cultured in DMEM (Gibco, Grand Island, NY, USA) supplemented with 10% fetal bovine serum (Gemini, Calabasas, CA, USA) and 1% penicillin-streptomycin (Gibco, Grand Island, NY, USA) at 37 °C with 5% CO_2_.

Bone marrow cells were isolated from DDX3X^fl/fl^ and DDX3X^ΔMφ^ mice. The femur and tibia of mice were isolated, the bone marrow cells were flushed out with pre-cooled DMEM medium, passed through a 70µm cell strainer, and centrifuged at 350g for 10 min. Then, the cells were incubated with Red Blood Cell Lysis Buffer (Beyotime, Shanghai, China) for 3 minutes at room temperature to lysate red blood cells. After termination of lysis, the cells were centrifuged at 350g for 10 min. The obtained bone marrow cells were cultured in complete DMEM medium containing 20ng/ml m-CSF (PeproTech, USA). After 3 days, the medium was changed, and the non-adherent cells were discarded, and the culture was continued for another 4 days to obtain differentiated mature bone marrow-derived macrophages (BMDMs). BMDMs were cultured in medium containing 10ug/ml LPS to promote its differentiation to M1 macrophages, and BMDMs were cultured in medium containing 20ng/ml IL-4 (PeproTech, USA) to promote its differentiation to M2 macrophages.

For the co-culture studies, primary hepatocytes from DDX3X^fl/fl^ or DDX3X^Δhep^ mice were cultured with BMDMs from DDX3X^fl/fl^ or DDX3X^ΔMφ^ mice respectively in the co-culture chamber (Corning, Arkansas, USA) for 24h under indicated treatment. Then BMDMs were collected for further flow cytometry analysis, and the hepatocytes were collected for the detection of lipid deposition.

**Biochemical analysis**

Serum alanine aminotransferase (ALT) was measured utilizing the ALT Activity Assay Kit (Cayman, Michigan, USA). Lipid peroxidation of liver tissue homogenates was determined by measuring malondialdehyde utilizing a TBARS Assay Kit (Cayman, Michigan, USA). The level of hepatic triglyceride (TG) was evaluated using a Triglyceride Colorimetric Assay Kit (Cayman, Michigan, USA) according to the manufacturer's instructions.

**Liver histology**

Liver tissue was fixed in paraformaldehyde, embedded in paraffin, and sectioned. Paraffin sections were dewaxed for hematoxylin and eosin (H&E) staining, and the degree of steatosis and lobular inflammation in the liver were evaluated by two pathologists independently according to NAS system[[1](#_ENREF_1), [2](#_ENREF_2)]. Liver lipid accumulation was further assessed by Oil Red O staining (Sigma Chemical, St Louis, MO) performed on frozen liver sections following the manufacturer's instructions[[3](#_ENREF_3)].

**Multiplexed immunohistochemical staining**

Multiplexed immunohistochemical staining was performed according to manufacturer's instruction by using multiplexed immunohistochemistry assay kit (Panovue, Beijing, China). In brief, paraffin-embedded liver sections were deparaffinized and the antigens were retrieved. The slides were covered in 3% hydrogen peroxide (H_2_O_2_) solution to abolish endogenous peroxidase activity, followed by blocked with 5% goat serum. Then the slides were incubated with primary antibodies at 4 °C overnight, followed by incubation with HRP-labeled secondary antibodies for 30 min. Signals were developed with monochromatic tyramide signal amplification (TSA) fluorescent dye, and the nuclei was presented with DAPI.

**Immunohistochemistry**

Paraffin sections of the liver tissues were dewaxed in xylene and hydrated in gradually decreasing concentrations of ethanol. Antigen retrieval was performed in citrate buffer at pH=6.0. Next, the slides were added with 3% hydrogen peroxide solution to inactivate endogenous peroxidase, and were blocked with goat serum at room temperature for 1 hour. Then the primary antibodies were incubated in a wet box at 4°C overnight. The corresponding primary antibodies were anti-F4/80 (ab235940, abcam, Cambridge, UK). The next day, the sections were incubated with secondary antibody for one hour at room temperature and stained with DAB solution (Zsbio, Beijing, China) for microscopic observation. Then, the nuclei were counterstained with hematoxylin. Finally, the slides were dehydrated, sealed and photographed.

**Immunofluorescence**

Cells were inoculated on glass coverslips and treated accordingly. Then cells were fixed with 4% paraformaldehyde at room temperature for 10 minutes. For protein targets located in the cytoplasm and nucleus, the cells were needed to be permeabilized with 0.25% Triton X-100 for 10 minutes. Then cells were blocked with 10% goat serum and incubated with primary antibodies in a humidified chamber overnight at 4°C. The antibodies were: DDX3X (ab235940, Abcam, Cambridge, UK), G3BP1 (CL488-66486, Proteintech, Chicago, USA), ASC (67824, Cell Signaling Technology, Boston, USA). The next day, the cells were incubated with secondary antibody for 1 hour at room temperature in the dark and the nuclei were stained with DAPI for 20 min at room temperature. For co-distribution of two different antigens in the same sample, it was performed sequentially. Finally, the coverslips were mounted with antifading mounting medium (InCellGene, Tx, USA) and visualized utilizing a confocal microscope (Nikon C2, Tokyo, Japan).

**Co-Immunoprecipitation (Co-IP)**

Primary hepatocytes were collected for further co-immunoprecipitation assay using a Pierce™ Co-Immunoprecipitation Kit (Thermo scientific, Waltham, USA). The antibody DDX3X (ab235940, Abcam, cambridge, UK) was used to pull down proteins that bind to it and the corresponding proteins were further detected by Western blotting.

**Fluorescence activated cell sorting (FACS) analysis**

BMDMs isolated from DDX3X^fl/fl^ and DDX3X^ΔMφ^ mice were used for FACS analysis. The cells were digested by trypsin and resuspend into a single cell suspension in Cell Staining Buffer (BioLegend, 420201, CA, USA). The Fc receptors of cells were blocked by CD16/32 (BioLegend, 156603, CA, USA)10 minutes at 4°C. The cell surface molecules were then stained at room temperature for 30 minutes in the dark, the corresponding antibodies were PE anti-mouse F4/80 antibody (BioLegend, 123109, CA, USA), FITC anti-mouse/human CD11b antibody (BioLegend, 101205, CA, USA), and PerCP/Cyanine5.5 anti-mouse I-A/I-E antibody (BioLegend, 107625, CA, USA). F4/80^+^CD11b^+^MHCII^+^ cells were identified as M1 macrophages. After cell surface antigen staining was finished, the cells were fixed in Fixation Buffer (FXP008, 4A Biotech, Beijing, China) 10 minutes at room temperature in the dark and permeablized in Permeabilization Wash Buffer (FXP009, 4A Biotech, Beijing, China) 15 minutes at room temperature in the dark for intracellular antigen staining. Cells were then stained with APC anti-mouse CD206 Antibody (BioLegend, 141707, CA, USA). F4/80^+^CD11b^+^CD206^+^ cells were identified as M2 macrophages. SA3800 Full Spectrum Flow Cytometer (Sony, Tokyo, Japan) were used for flow cytometry assay and FlowJo software (CA, USA) were utilized for analysis.

**SYTOX Green nucleic acid staining**

SYTOX (MX4228, Maokangbio, Shanghai, China) was diluted in DMEM medium to a final concentration of 1μM. Cells were incubated in working solution for 1 h and washed in darkness. Then cells were fixed in 4% paraformaldehyde for 10 minutes and stained with DAPI in darkness for 20 minutes at room temperature.

**LDH release assay**

Cells were seeded into 96-well cell culture plates to a density of 90% confluence. LDH content released by cells under different treatments was detected following the manufacturer's instructions (C0016, Beyotime, Shanghai, China).

**RNA extraction and qRT-PCR**

Total RNA from liver tissues and primary cells was isolated with Takara MiniBEST Universal RNA Extraction Kit (Takara, Kyoto, Japan). cDNA was synthesized by using Takara PrimeScript RT reagent Kit (Takara, Kyoto, Japan). qRT-PCR was conducted using SYBR Green qPCR MasterMix (abm, VAN, Canada). Detail of the primer sequences were shown in ***Table. S1***.

**Western blotting**

Tissues and cells were lysed by RIPA lysis buffer (Beyotime, Shanghai, China) containing protease inhibitor cocktail and phosphatase inhibitor. Total protein was quantified using BCA Protein Assay Kit (Thermo Fisher Scientific, MA, USA). Equal amounts of protein were added to SDS-PAGE gels for electrophoresis, transferred to NC membranes, blocked with 5% skim milk, and incubated with a suitable concentration of primary antibody overnight at 4°C. The involved antibodies were: DDX3X (ab235940, abcam, cambridge, UK), G3BP1 (13057-2-AP, Proteintech, Chicago, USA), NLRP3 (ab263899, abcam, cambridge, UK), ASC (67824, Cell Signaling Technology, Boston, USA), NLRC4 (A7382, ABclonal, Wuhan, China), GSDMD (ab209845, abcam, cambridge, UK), IL-1β (63124, Cell Signaling Technology, Boston, USA). After incubation of the corresponding secondary antibodies, the protein was visualized with UltraSignal hypersensitive ECL chemiluminescence substrate (4A Biotech, Beijing, China).

**Statistical Analysis**

All experiments were repeated at least three times with consistent results. Statistical analysis was performed using the statistical software package SPSS 13.0 (SPSS, Chicago, Illinois, USA) and quantitative data were expressed as mean ± SD. All tests of significance were two-sided and *p* values< 0.05 were considered statistically significant.

**Supplementary References**

1. Sanyal AJ, Brunt EM, Kleiner DE, Kowdley KV, Chalasani N, Lavine JE, et al. Endpoints and clinical trial design for nonalcoholic steatohepatitis. Hepatology. 2011; 54: 344-53.

2. Kleiner DE, Brunt EM, Van Natta M, Behling C, Contos MJ, Cummings OW, et al. Design and validation of a histological scoring system for nonalcoholic fatty liver disease. Hepatology. 2005; 41: 1313-21.

3. Zhang X, Shen J, Man K, Chu ES, Yau TO, Sung JC, et al. CXCL10 plays a key role as an inflammatory mediator and a non-invasive biomarker of non-alcoholic steatohepatitis. J Hepatol. 2014; 61: 1365-75.

**Supplementary Figures**

**
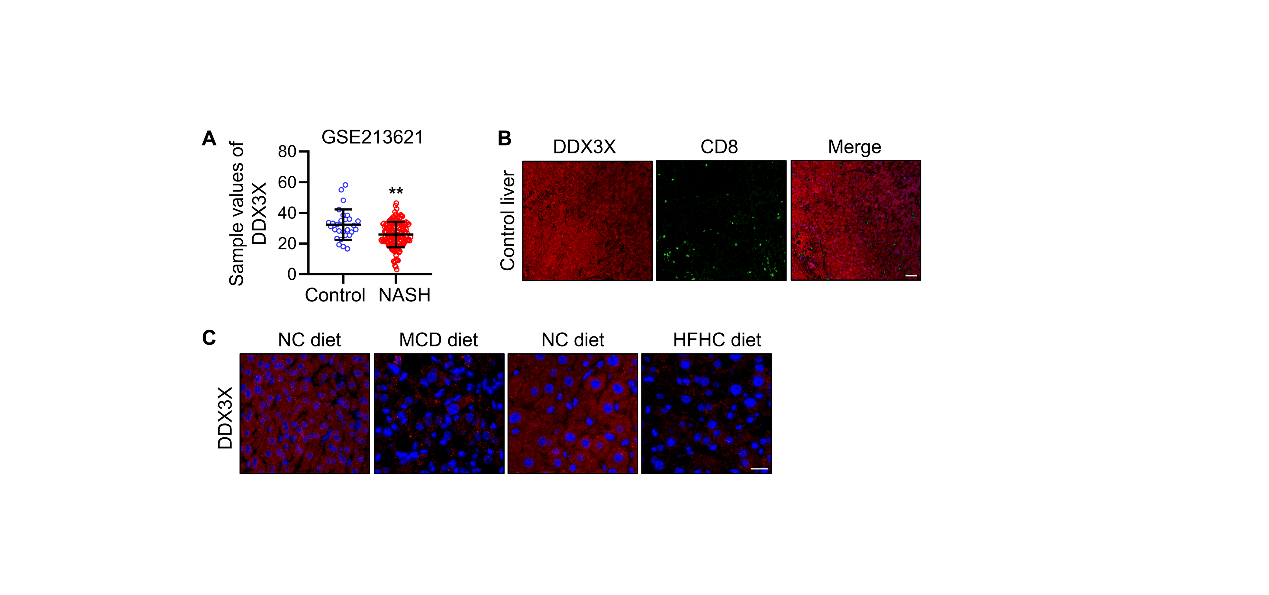
**

**Fig. S1 Expression and distribution of DDX3X in liver tissues of human and mice.** (A) The expression of DDX3X in liver tissues of NASH patients (n=151) and control liver tissues (n=28) in GSE213621. Data are expressed as mean ± SD. ***p*< 0.01. (B) Representative multiplexed immunohistochemical stained sections of DDX3X in CD8^+^T cells of human liver tissues. Scale bars, 50µm. (C) Representative immunofluorescence stained sections of DDX3X in nutritional mouse models of steatohepatitis fed with a HFHC diet or an MCD diet. Scale bars, 20µm.


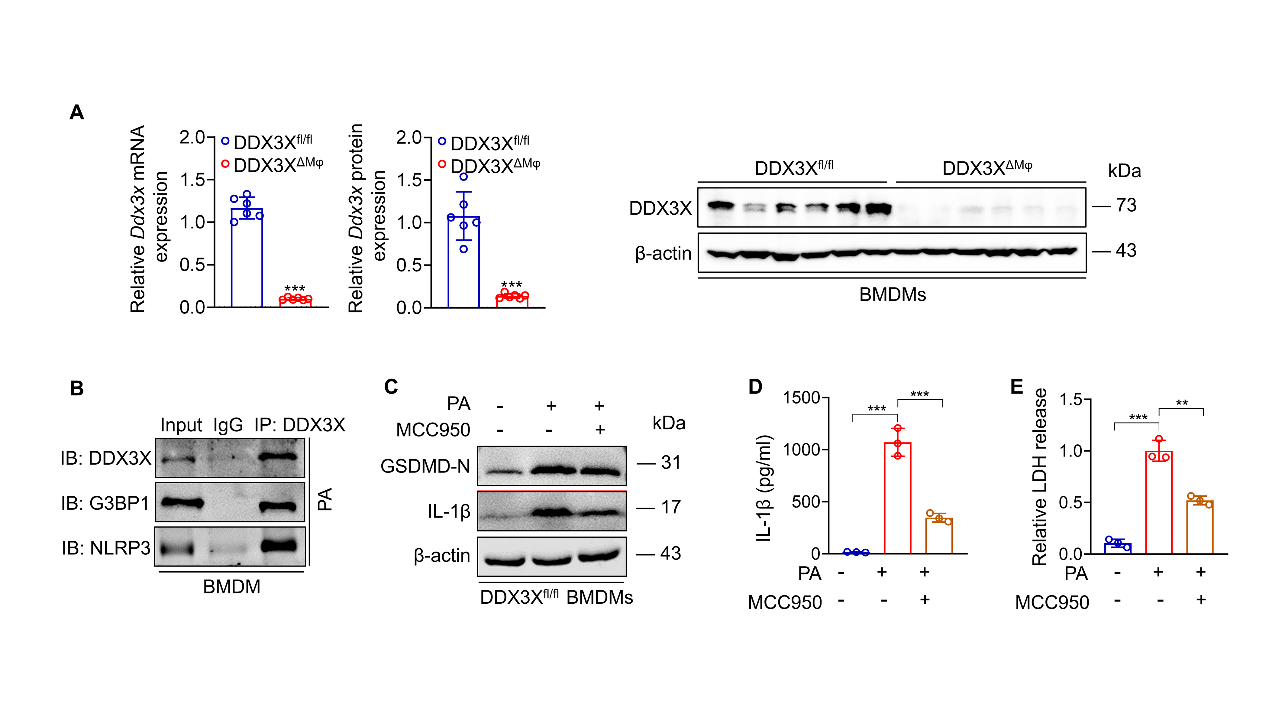


**Fig. S2 DDX3X induces NLRP3 inflammasome activation and pyroptosis in BMDMs treated with PA. (A)** *Ddx3x* mRNA and protein expression levels of BMDMs isolated from DDX3X^fl/fl^ and DDX3X^ΔMφ^ mice. **(B)** Co-IP experiments were performed to determine the interaction of DDX3X with G3BP1 and NLRP3 under PA induction (0.4 μM, 24 hours)in BMDMs. **(C)** GSDMD-N and IL-1β levels, **(D)** IL-1β release, and **(E)** medium LDH levels in BMDMs isolated from DDX3X^fl/fl^ mice treated with or without MCC950 under PA induction (0.4 μM, 24 hours). Data are expressed as mean ± SD, n=3-6/group. ***p*< 0.01, ****p*<0.001.


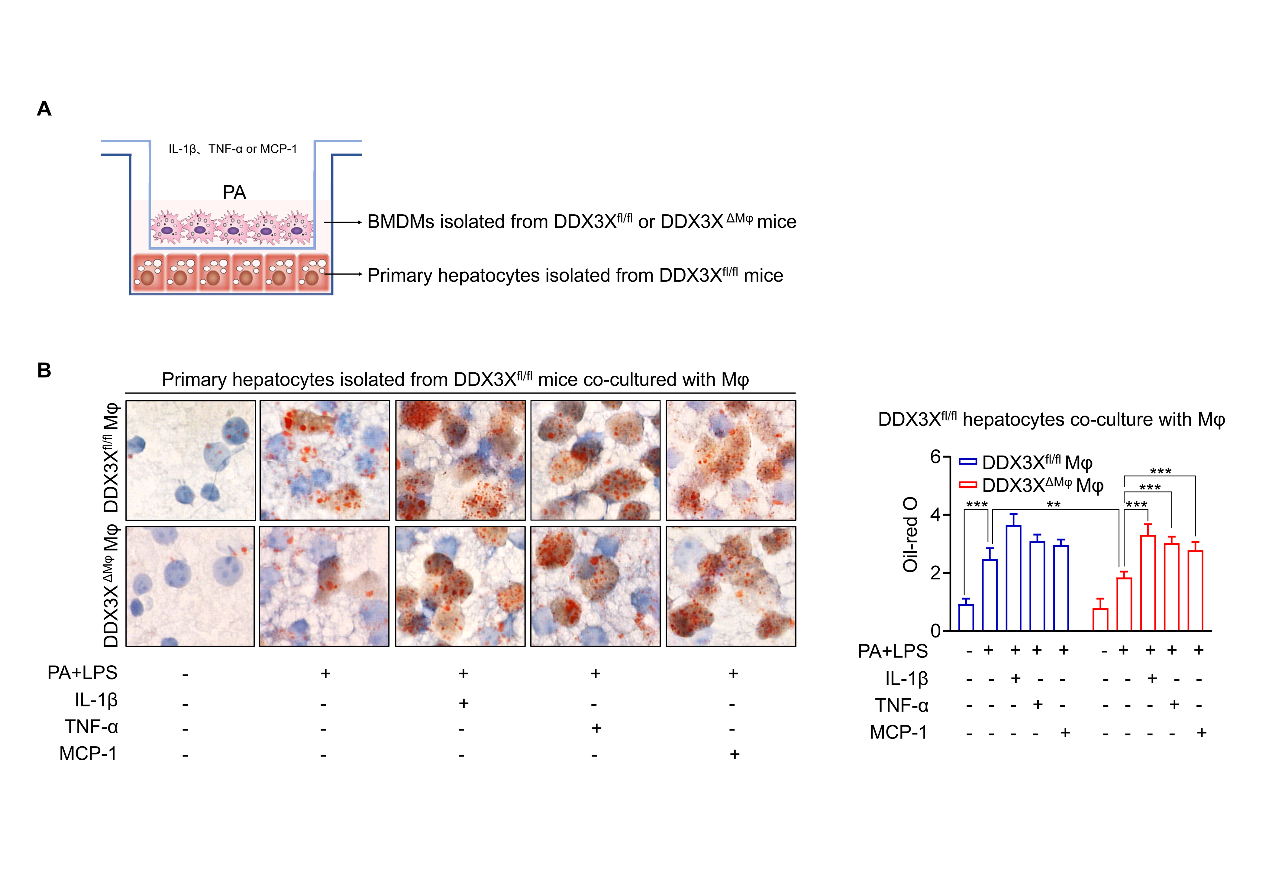


**Fig. S3 Macrophage DDX3X causes pro-inflammatory cytokines production and hepatocyte steatohepatitic changes. (A)** Schematic diagram of DDX3X^fl/fl^ or DDX3X^ΔMφ^ macrophages co-cultured with DDX3X^fl/fl^ hepatocytes. **(B)** Representative Oil-red O-stained sections of DDX3X^fl/fl^ hepatocytes co-cultured with DDX3X^fl/fl^ or DDX3X^ΔMφ^ macrophages in PA+LPS medium supplemented with TNF-α, IL-1β or MCP-1. Scale bars, 10µm. Data are expressed as mean ± SD, n=3/group. ****p*<0.001.


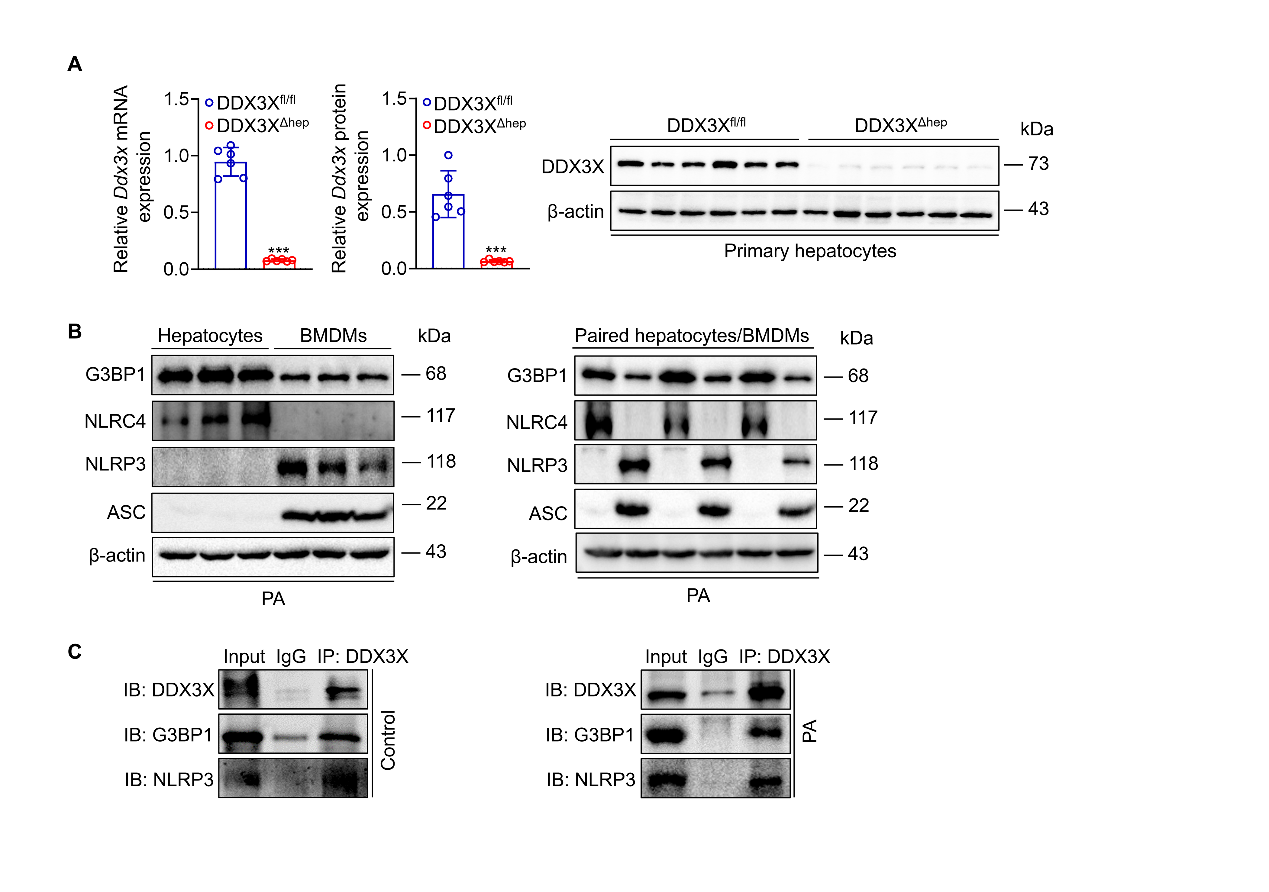


**Fig. S4** **The roles of hepatocyte DDX3X in SG assembly, NLRP3 and NLRC4 inflammasome activation. (A)** *Ddx3x* mRNA and protein expression in primary hepatocytes isolated from DDX3X^fl/fl^ and DDX3X^Δhep^ mice. Data are expressed as mean ± SD, n=6/group. ****p*<0.001. **(B)** Expression and distribution of G3BP1, ASC, NLRP3 and NLRC4 in primary hepatocytes and BMDMs isolated from DDX3X^fl/fl^ mice stimulated *in vitro* with PA (0.4 μM, 24 hours). **(C)** Co-IP experiments were performed to determine the interaction of DDX3X with G3BP1 and NLRP3 in primary hepatocytes.


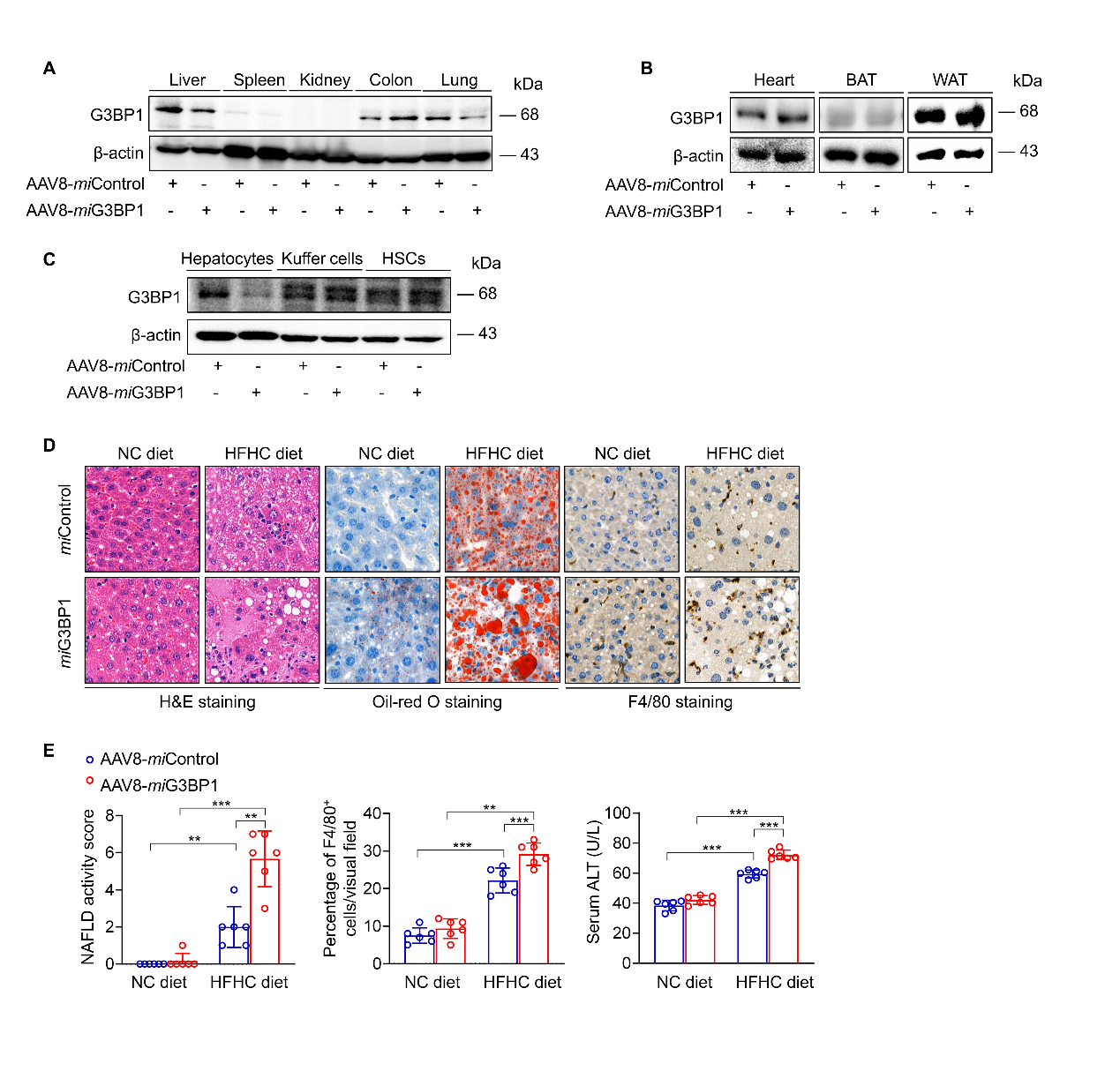


**Fig. S5 Deficiency of hepatocyte G3BP1 exacerbates experimental** **steatohepatitis in mice. (A-C)** Expression and distribution of G3BP1 in major organs, tissues or primary cells when mice injecting with AAV8-*mi*G3BP1 or AAV8-*mi*Control adeno-associated virus containing the liver-specific promoter TBG for 2 weeks. **(D)** Histological evaluation of liver sections from HFHC-fed mice injecting with AAV8-*mi*G3BP1 or AAV8-*mi*Control adeno-associated virus. Scale bars, 20µm. **(E)** Serum ALT levels, NAFLD activity score and macrophage infiltration of HFHC-fed mice injecting with AAV8-*mi*G3BP1 or AAV8-*mi*Control adeno-associated virus. Data are expressed as mean ± SD, n=6/group. ***p*< 0.01, ****p*<0.001.


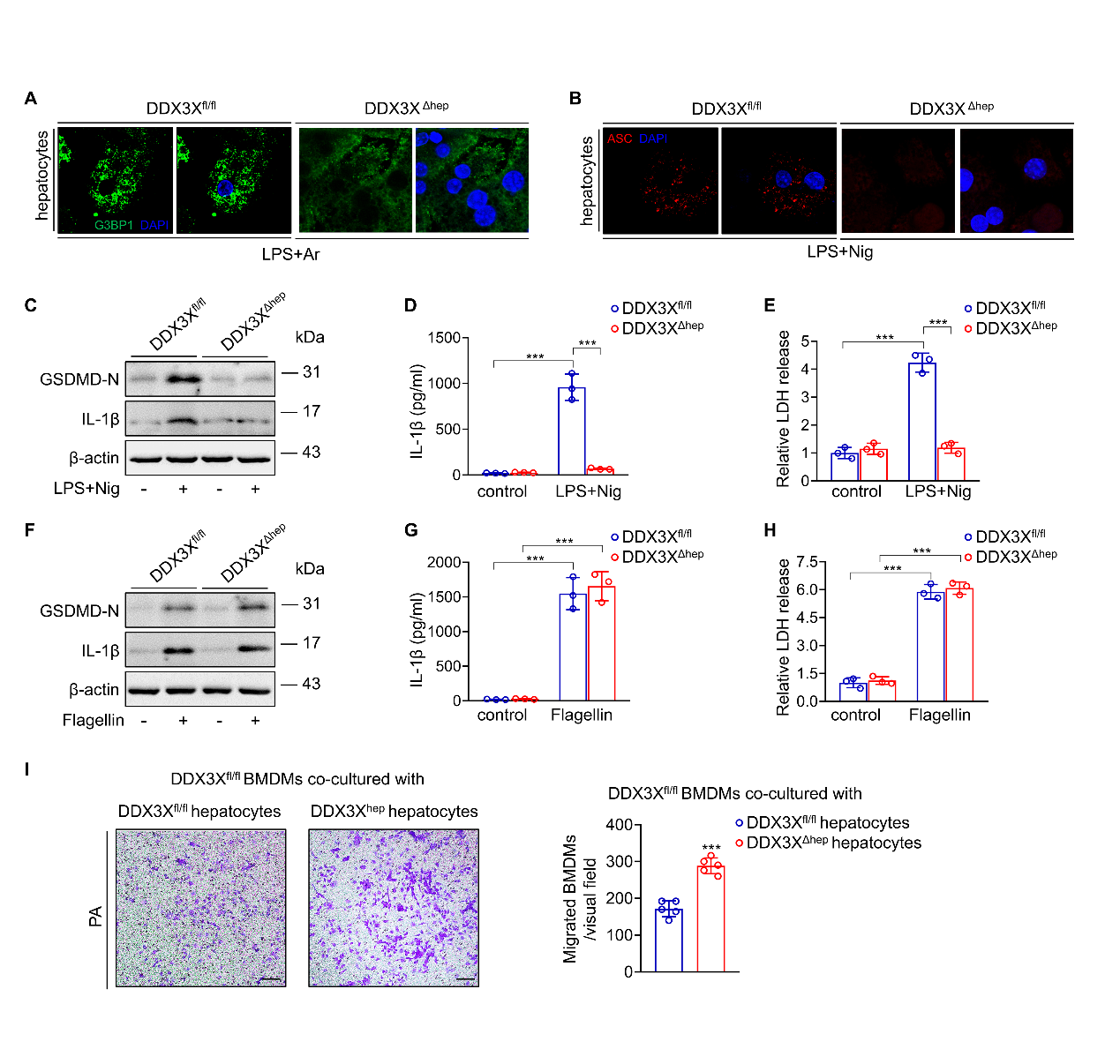


**Fig. S6 Hepatocyte DDX3X deletion causes defects in SG assembly and NLRP3 inflammasome activation but fails to protect against NLRC4-mediated hepatocyte pyroptosis. (A)** Representative IF images of G3BP1 in primary hepatocytes isolated from DDX3X^fl/fl^ or DDX3X^Δhep^ mice primed with LPS for 4 hours and then stimulated with the SGs inducer arsenite (Ar, 50 μM) for 30 minutes. Scale bars, 10µm. **(B)** Representative IF images of ASC, **(C)** GSDMD-N and IL-1β protein levels, **(D)** IL-1β release, and **(E)** medium LDH levels in primary hepatocytes isolated from DDX3X^fl/fl^ or DDX3X^Δhep^ mice primed with LPS for 4 hours and then stimulated with the NLRP3 stimulus nigericin (Nig, 20 μM) for 45 minutes. Scale bars, 10µm. **(F)** GSDMD-N and IL-1β protein levels, **(G)** IL-1β release, and **(H)** medium LDH levels in primary hepatocytes isolated from DDX3X^fl/fl^ or DDX3X^Δhep^ mice transfected with the NLRC4 stimulus flagellin for 4 hours. (I) The migrated DDX3X^fl/fl^ macrophages cocultured with DDX3X^fl/fl^ hepatocytes or DDX3X^Δhep^ hepatocytes under PA induction (0.4 μM, 24 hours). Scale bars, 200µm. Data are expressed as mean ± SD, n=3/group. ****p*<0.001.

**Table S1. Sequences of primers and interference sequences of siRNA**

| **Gene** | **Sequence** | **Description** |
| --- | --- | --- |
| *Ddx3x* (mouse) | CTATGCCTCCAAAAGGTGTCCG | Forward primer |
|  | AGACCCAACTCTTCCTACAGCC | Reverse primer |
| *Il-1β* (mouse) | TCAGGCAGGCAGTATCACTCATT | Forward primer |
|  | GGAAGGTCCACGGGAAAGA | Reverse primer |
| *Tnf-α* (mouse) | CGTGCTCCTCACCCACAC | Forward primer |
|  | GGGTTCATACCAGGGTTTGA | Reverse primer |
| *Mcp-1* (mouse) | CAAAATGAGCCGCAGCACCAGAAT | Forward primer |
|  | AGGCAGGCGCGGCAGAGACG | Reverse primer |
| *Il-10* (mouse) | CGGGAAGACAATAACTGCACCC | Forward primer |
|  | CGGTTAGCAGTATGTTGTCCAGC | Reverse primer |
| *β-actin* (mouse) | CATCCGTAAAGACCTCTATGCCAAC | Forward primer |
|  | ATGGAGCCACCGATCCACA | Reverse primer |
| *Nlrp3* (mouse) | CCAGGAGAGAACCUCUUAUTT | Si-*Nlrp3*-1 sense |
|  | AUAAGAGGUUCUCUCCUGGTT | Si-*Nlrp3*-1 antisense |
| *Nlrp3* (mouse) | GGACCUCAGUGACAAUACUTT | Si-*Nlrp3*-2 sense |
|  | AGUAUUGUCACUGAGGUCCTT | Si-*Nlrp3*-2 antisense |
| *Nlrc4* (mouse) | CCCGAAAGAUCAUCCAUAUTT | Si-*Nlrc4*-1 sense |
|  | AUAUGGAUGAUCUUUCGGGTT | Si-*Nlrc4*-1 antisense |
| *Nlrc4* (mouse) | GGUGAAGAUAUCGACAUAATT | Si-*Nlrc4*-2 sense |
|  | UUAUGUCGAUAUCUUCACCTT | Si-*Nlrc4*-2 antisense |

**Western bolts data**


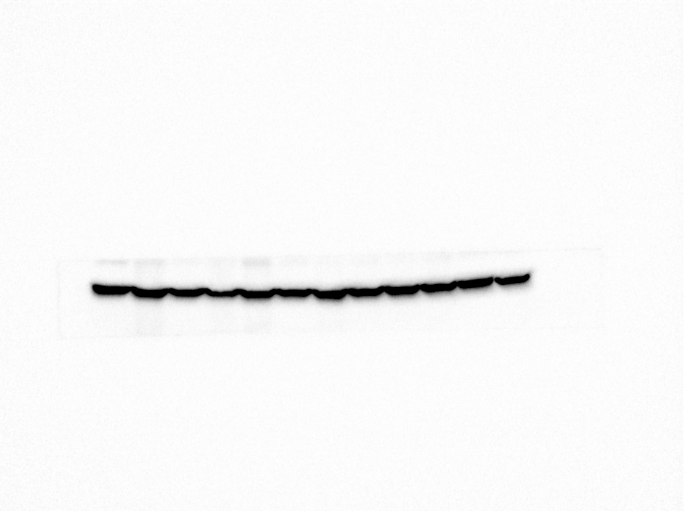


**Figure. 1B β-actin** (Livers of mice fed with NC or HFHC diet)


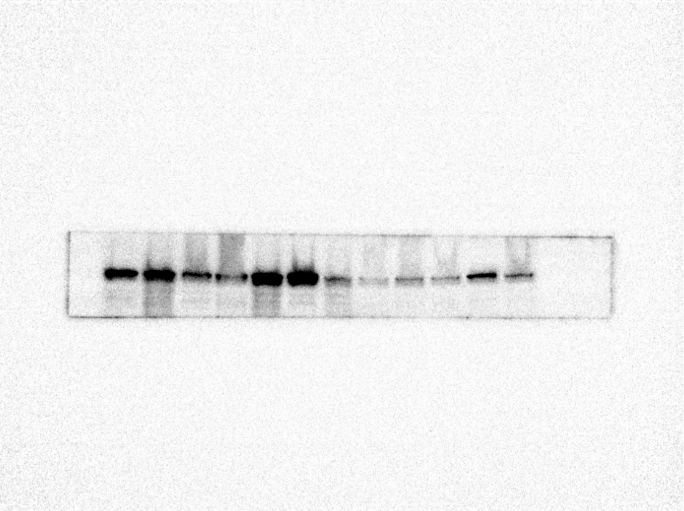


**Figure. 1B DDX3X** (Livers of mice fed with NC or HFHC diet)


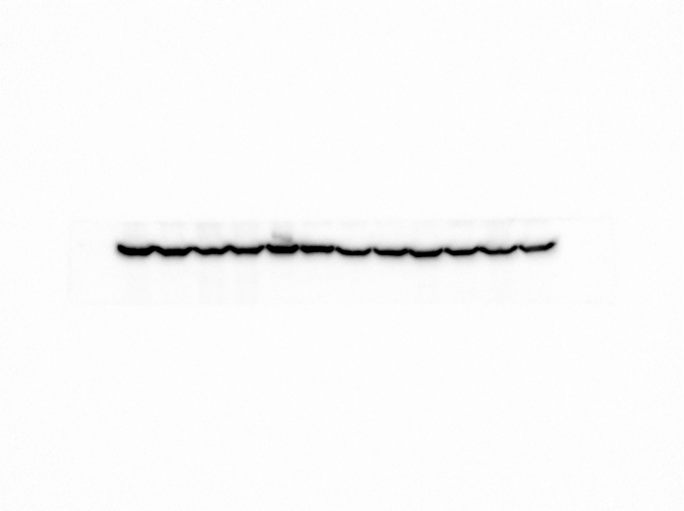


**Figure. 1B β-actin** (Livers of mice fed with NC or MCD diet)


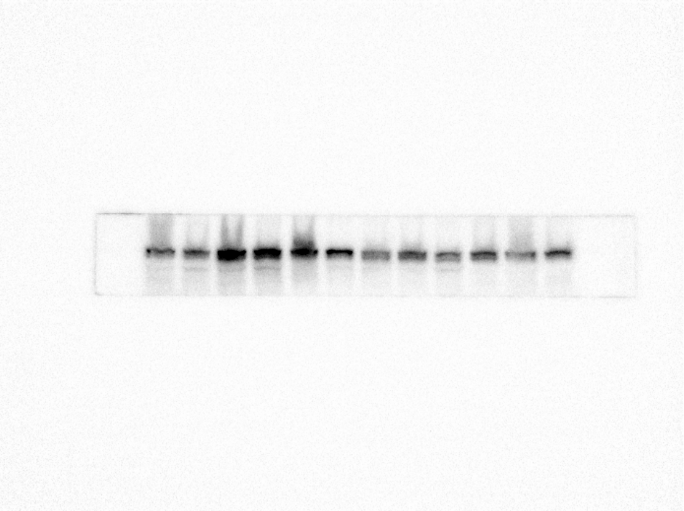


**Figure. 1B DDX3X** (Livers of mice fed with NC or MCD diet)


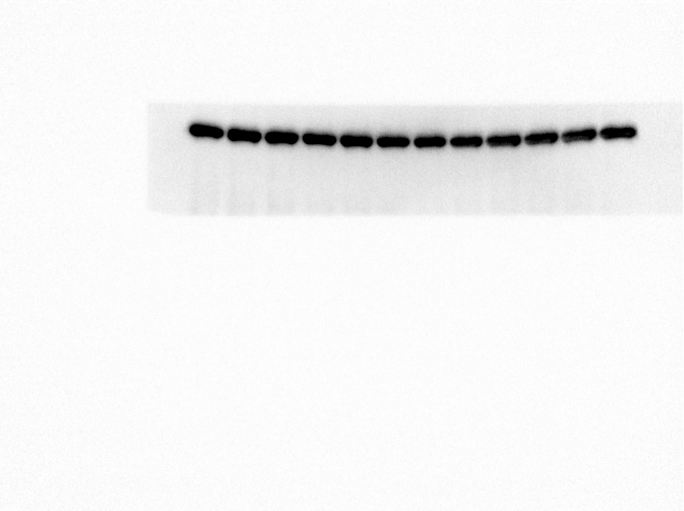


**Figure. 1B β-actin** (Livers of mice fed with NC or HFHIHFHC-MCD diet)


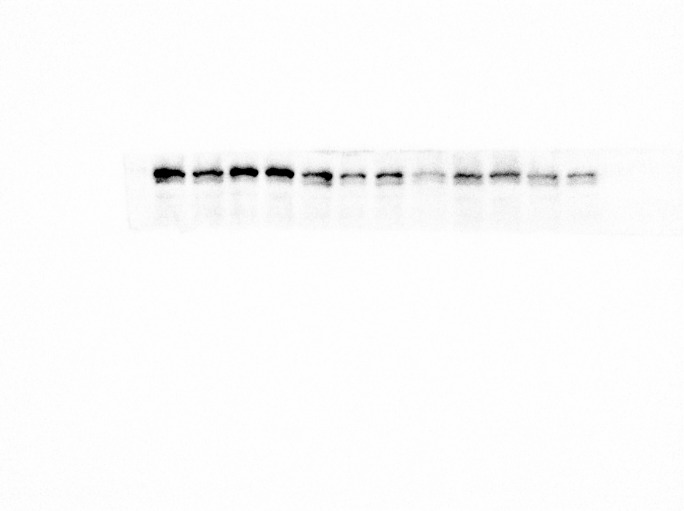


**Figure. 1B DDX3X** (Livers of mice fed with NC or HFHIHFHC-MCD diet)


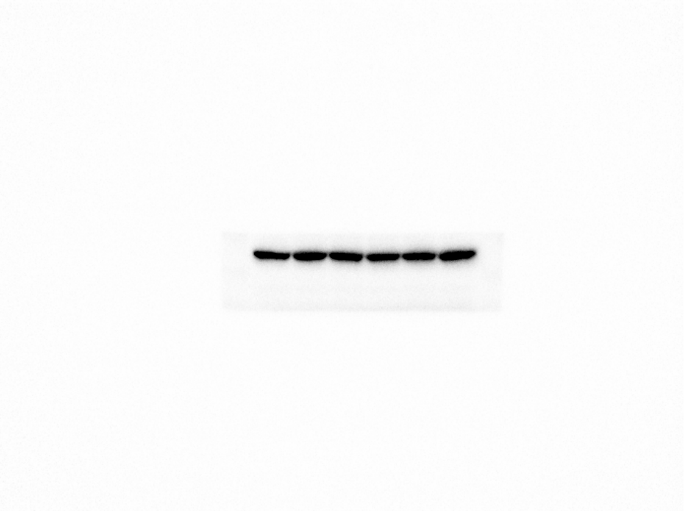


**Figure. 1C β-actin** (Primary hepatocytes and BMDMs isolated from DDX3X^fl/fl^ mice)


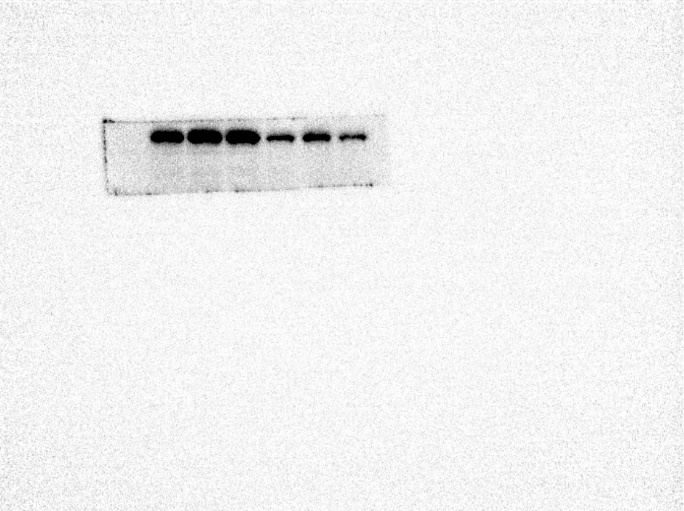


**Figure. 1C DDX3X** (Primary hepatocytes and BMDMs isolated from DDX3X^fl/fl^ mice)


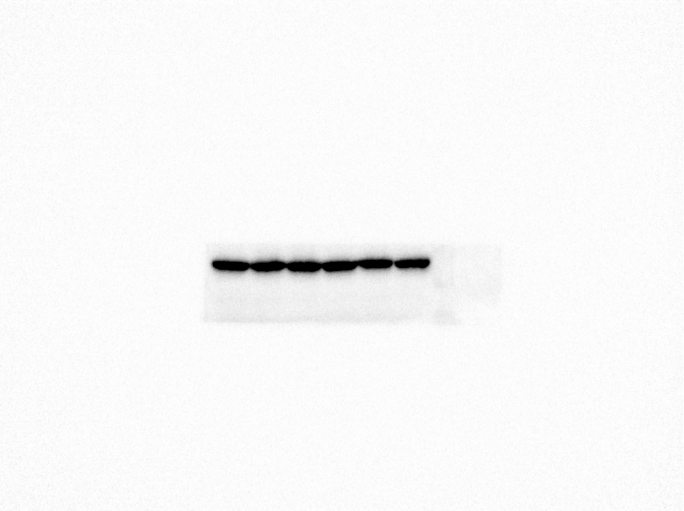


**Figure. 1D β-actin** (Primary hepatocytes and BMDMs isolated from DDX3X^fl/fl^ mice-Paired)


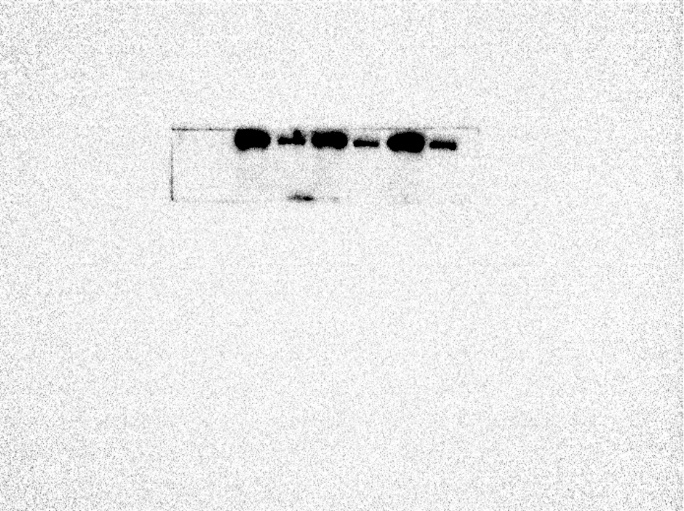


**Figure. 1D DDX3X** (Primary hepatocytes and BMDMs isolated from DDX3X^fl/fl^ mice-Paired)


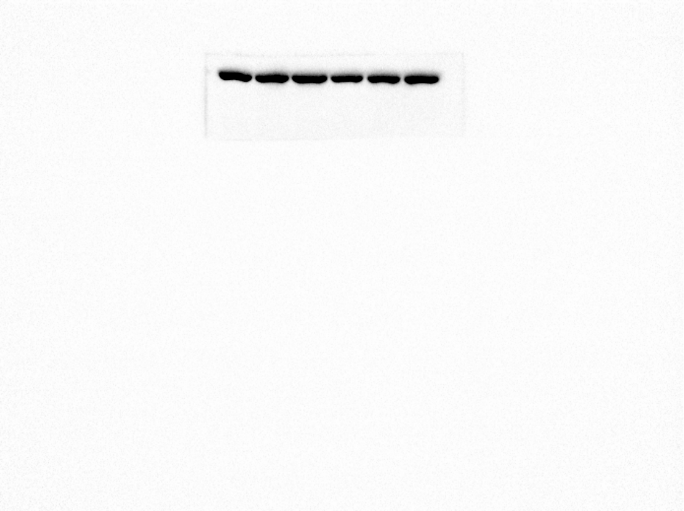


**Figure. 1E β-actin** (Primary hepatocytes treated with control or PA medium)


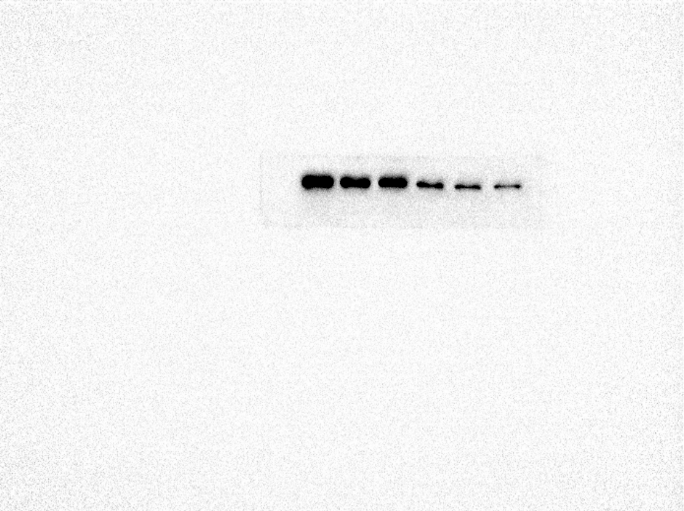


**Figure. 1E DDX3X** (Primary hepatocytes treated with control or PA medium)


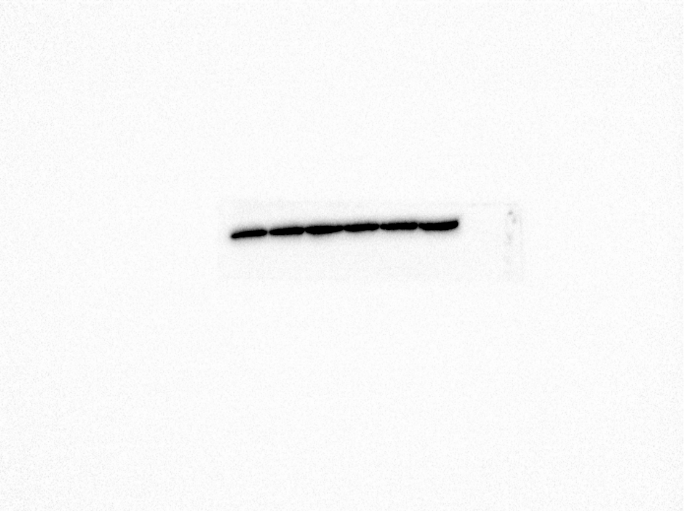


**Figure. 1F β-actin** (BMDMs treated with control or PA medium)


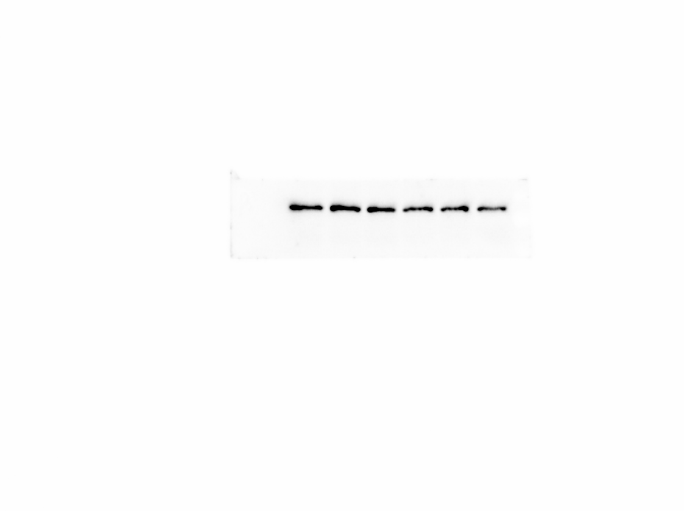


**Figure. 1F DDX3X** (BMDMs treated with control or PA medium)


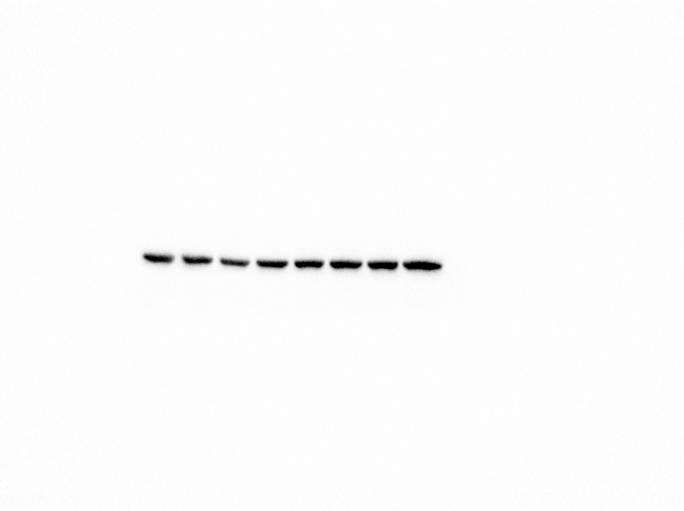


**Figure. 3F β-actin**





**Figure. 3F IL-1β**


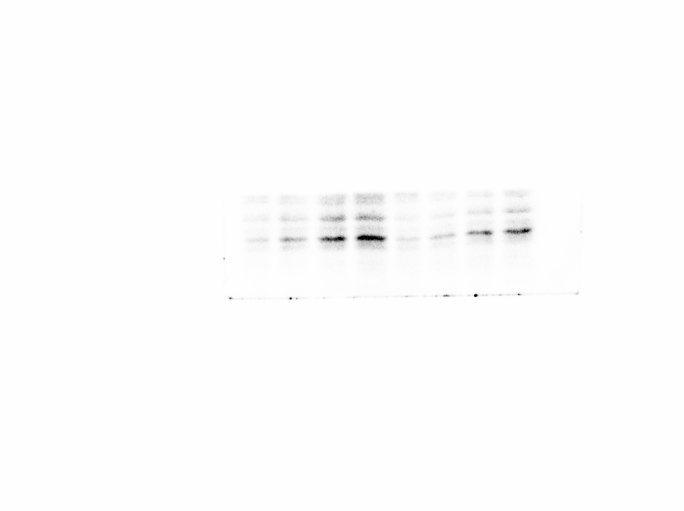


**Figure. 3F GSDMD-N**





**Figure. 3H β-actin**





**Figure. 3H IL-1β**





**Figure. 3H GSDMD-N**


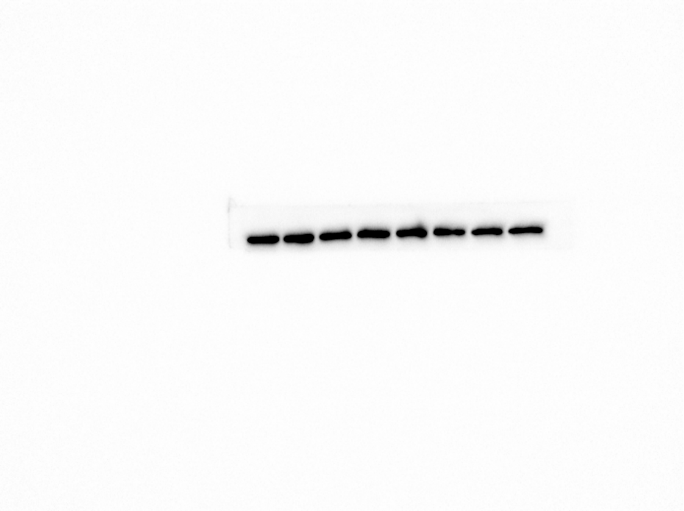


**Figure. 6F β-actin**





**Figure. 6F IL-1β**


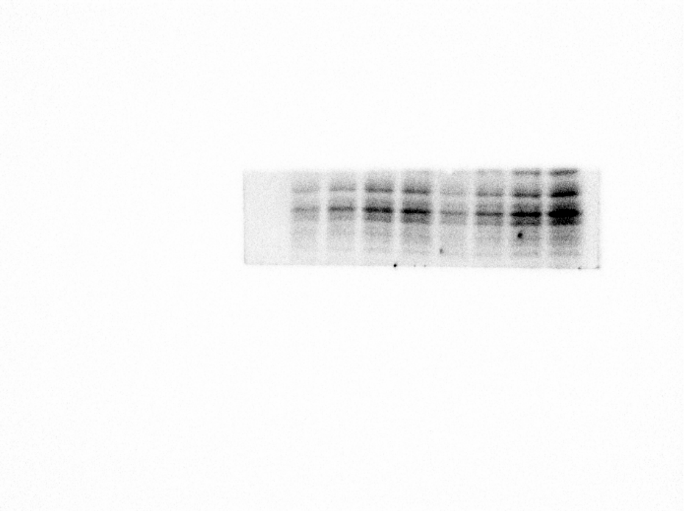


**Figure. 6F GSDMD-N**


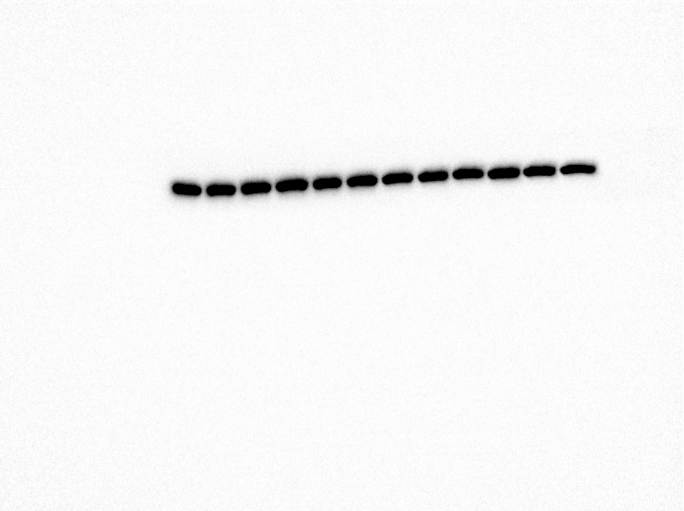


**Figure. 6G β-actin (**Primary hepatocytes isolated from DDX3X^fl/fl^ or DDX3X^Δhep^ mice**)**


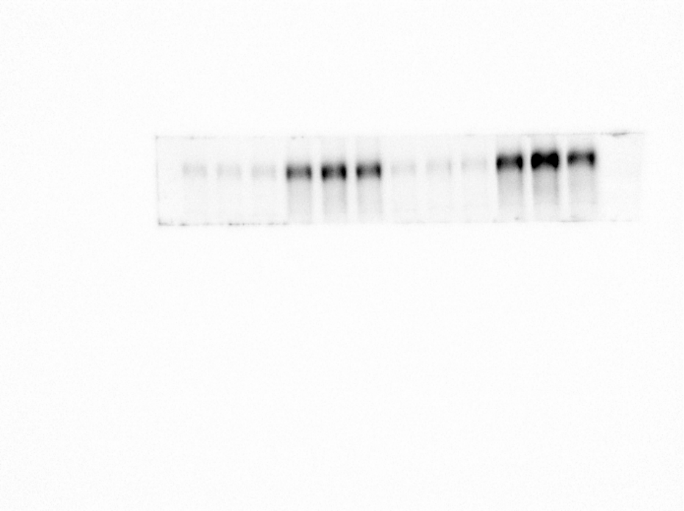


**Figure. 6G NLRC4 (**Primary hepatocytes isolated from DDX3X^fl/fl^ or DDX3X^Δhep^ mice**)**


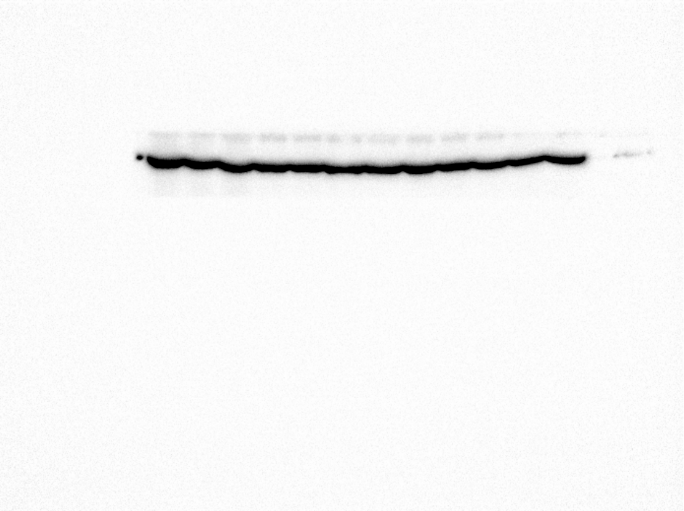


**Figure. 6G β-actin (**Liver tissues from DDX3X^fl/fl^ or DDX3X^Δhep^ mice**)**


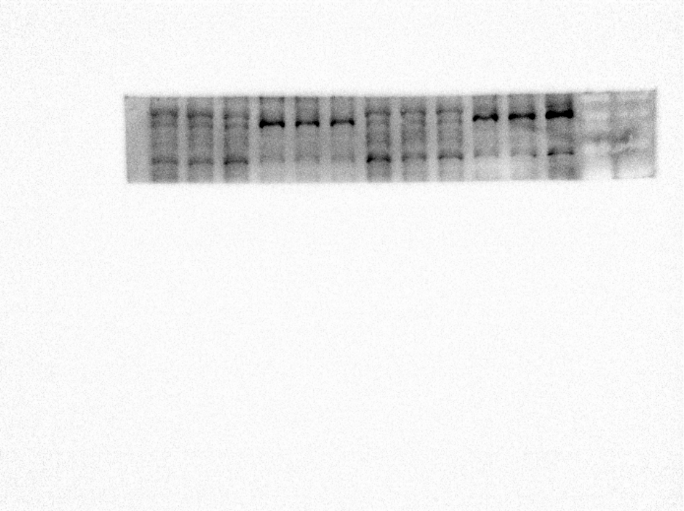


**Figure. 6G NLRC4 (**Liver tissues from DDX3X^fl/fl^ or DDX3X^Δhep^ mice**)**





**Figure. 6H β-actin**





**Figure. 6H IL-1β**





**Figure. 6H GSDMD-N**
